# ER-associated degradation in cystinosis pathogenesis and the prospects of precision medicine

**DOI:** 10.1172/JCI169551

**Published:** 2023-10-02

**Authors:** Varsha Venkatarangan, Weichao Zhang, Xi Yang, Jess Thoene, Si Houn Hahn, Ming Li

**Affiliations:** 1 Department of Molecular, Cellular, and Developmental Biology, University of Michigan, Ann Arbor, Michigan, USA.; 2 Department of Pediatrics, Division of Pediatric Genetics, Metabolism & Genomic Medicine, University of Michigan School of Medicine, Ann Arbor, Michigan, USA.; 3 University of Washington School of Medicine, Department of Pediatrics, Seattle Children’s Hospital, Seattle, Washington, USA.

**Keywords:** Cell Biology, Nephrology, Chronic kidney disease, Genetic diseases, Ubiquitin-proteosome system

## Abstract

Cystinosis is a lysosomal storage disease that is characterized by the accumulation of dipeptide cystine within the lumen. It is caused by mutations in the cystine exporter, cystinosin. Most of the clinically reported mutations are due to the loss of transporter function. In this study, we identified a rapidly degrading disease variant, referred to as cystinosin(7Δ). We demonstrated that this mutant is retained in the ER and degraded via the ER-associated degradation (ERAD) pathway. Using genetic and chemical inhibition methods, we elucidated the roles of HRD1, p97, EDEMs, and the proteasome complex in cystinosin(7Δ) degradation pathway. Having understood the degradation mechanisms, we tested some chemical chaperones previously used for treating CFTR F508Δ and demonstrated that they could facilitate the folding and trafficking of cystinosin(7Δ). Strikingly, chemical chaperone treatment can reduce the lumenal cystine level by approximately 70%. We believe that our study conclusively establishes the connection between ERAD and cystinosis pathogenesis and demonstrates the possibility of using chemical chaperones to treat cystinosin(7Δ).

## Graphical abstract

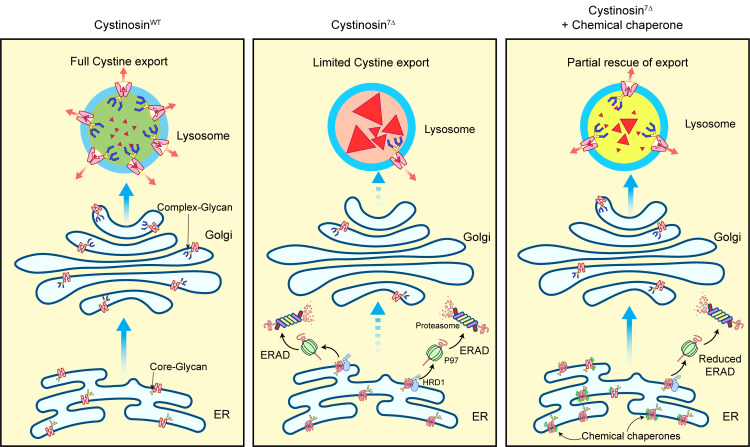

## Introduction

There are myriad pathogenesis mechanisms associated with human genetic disorders. The mutated proteins either (a) lose or gain function, (b) lose or gain protein stability, (c) suffer trafficking defects, or (d) suffer changes in expression level. Therefore, understanding the unique mechanism of each patient’s mutation is monumentally important, as it will determine their corresponding clinical therapy. For instance, cystic fibrosis, the most common and fatal genetic disease in the US, has an array of clinically reported mutations in the cystic fibrosis transmembrane conductance regulator (CFTR), a chloride channel on the cell membrane. While many of these mutations lead to the loss of transport function, the most common mutation, a deletion of phenylalanine (F) at residue 508 (ΔF508), causes cystic fibrosis due to its rapid degradation at the ER (1). Chemically stabilized CFTR ΔF508 is shown to be functional and can ameliorate the disease progression (2). Another example is Niemann Pick disease type c, wherein the lysosomal lipid transporter NPC1 is mutated, leading to a lysosomal buildup of cholesterol. Several mutations in NPC1 cause the loss of lipid transporting ability, but the most frequent mutation, I1061T, leads to the disease due to its proteolysis at both the ER and the lysosomes (3, 4).

Intrigued by the role of erroneous protein degradation in genetic diseases (5), we aimed to explore this aspect in the pathogenesis of a devastating lysosomal storage disease (LSD), cystinosis (6–8). Cystinosis is a recessive genetic disease caused by mutations in cystinosin, the cystine exporter on the lysosomal membrane (9, 10). The cystine transport from the lumen to the cytosol prevents the buildup of the amino acid within the lysosome (11). It also plays a vital role in antioxidant glutathione synthesis (12) and autophagy maintenance by preventing mTORC1 reactivation during starvation (13). In untreated patients, the kidneys and eyes are the first organs to be affected (14). The disease progression leads to complete renal failure within the first ten years of age. Patients also suffer from hypothyroidism, weakened bones and muscles, and reduced cognitive abilities (15, 16).

At the protein level, cystinosin is a 7-transmembrane protein that belongs to the PQ (P- proline, Q- glutamine) loop family of transporters. It contains 367 amino acids with an N-terminal signal peptide and a C-terminal tyrosine motif for lysosome targeting. Additionally, the second PQ motif is also involved in the lysosomal targeting (17). The transporter is heavily N-link glycosylated with 7 putative glycosylation sites in the lumenal loop (9). The recent structural work by Guo et al. and Löbel et al. (18, 19). identified many residues critical for the transporter functioning. Their discoveries are well corroborated with the sequenced patient database. Nevertheless, it is important to note that there are disease-causing mutations that are not directly involved in the transport function. We hypothesized that some of these mutations could likely cause the disease due to protein degradation rather than loss of function.

To analyze this further, we screened through different clinically reported cystinosin mutations to search for rapidly degraded mutants and identified a mutant that harbors a deletion of 7 amino acids (the residues I, T, I, L, E, L, and P) from positions 67 to 73. Hereafter, we will refer to this mutant as cystinosin(7Δ) or 7Δ in short. Previous studies have reported that 7Δ has a glycosylation defect due to the disruption of the N^^66^^xT^^68^^ site. It was also suggested that cystinosin(7Δ) traffics to the lysosome and then is degraded at the organelle through an unknown mechanism (20).

Through a careful investigation, we demonstrated that most 7Δ is actually trapped in the ER, ubiquitinated by the E3 ligase HRD1, and is degraded by the ER-associated degradation (ERAD) pathway. The glycosylation defect is due to its inability to exit the ER, instead of the disruption of the N66 glycosylation site. We further tested several small molecule chemical chaperones, some of which have been FDA-approved, for the treatment CFTR ΔF508 (21, 22). We demonstrated that 1 of the chemical chaperones (corr-4a) reduced the degradation of cystinosin(7Δ) and facilitated its trafficking to the lysosome. Importantly, the lysosome-targeted cystinosin(7Δ) is functional as it relieves the accumulated lysosomal cystine levels by 70% in patient fibroblast cells. Collectively, our work establishes the role of ERAD in cystinosis pathogenesis and identifies potential chemical chaperones that could serve as precision medicine for this mutation.

## Results

### A cycloheximide chase screen to assay the half life of cystinosin disease mutants.

**Figure 1:**
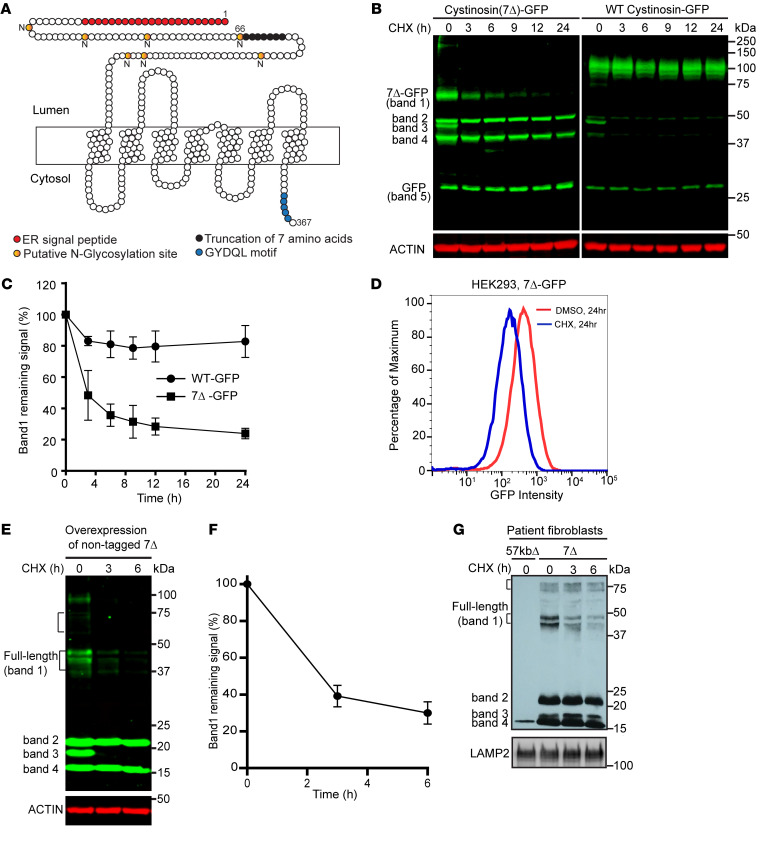
Identification of cystinosin(7Δ) as a rapidly degrading disease variant. (**A**) Topology map of cystinosin: cystinosin is a 7-transmembrane protein that contains a large lysosomal lumenal loop, 7 putative N-linked glycosylation sites (gold), an N-terminal signal peptide (red) and a C-terminal lysosome targeting motif (blue). The 7 amino acid deletion in Cystinosin(7Δ) is shown in black. (**B**) CHX chase was performed in cells stably expressing either cystinosin(7Δ)-GFP or WT cystinosin-GFP. Cystinosin-GFP appears predominantly as a band of apprpximately 100 kDa, which remains stable throughout the chase. Meanwhile, cystinosin(7Δ)-GFP is present as 4 bands and a free GFP band. Bands 1 and 3 degrade rapidly, whereas bands 2 and 4 are stable. (**C**) Quantification of the full-length cystinosin(7Δ)-GFP (band 1) and WT cystinosin-GFP in **B**. (**D**) Flow cytometry measurement of cystinosin(7Δ)-GFP fluorescence signal after 24 hours of CHX treatment. (**E**) CHX chase experiment in cells stably expressing untagged cystinosin(7Δ), probed with an endogenous cystinosin antibody. The bracket at approximately 75kDa highlights a small population of higher molecular weight species. (**F**) Quantification of band 1 in **E**. (**G**) CHX chase performed in patient fibroblasts homozygous for 7Δ. To ensure antibody specificity, a control fibroblast from a patient with CTNS deletion (57 kb genomic DNA deletion) was included. The bracket at approximately 75kDa highlights a small population of higher molecular weight species. Error bars indicate the SD. All quantifications and data analysis were performed based on 3 independent replicates.

To test if any cystinosin mutants could be constitutively degraded, we generated a plasmid library by fusing 37 disease-causing CTNS mutations with a c-terminal GFP tag. We then transiently overexpressed these constructs in HEK293 cells and performed a cycloheximide (CHX) chase assay for up to 24 hours to measure their protein stability (Supplemental Figure 1; supplemental material available online with this article; https://doi.org/10.1172/JCI169551DS1). We identified several slow-degrading mutants. In addition, we also isolated one fast-degrading mutation that contained a truncation of 7 amino acids (__67__ITILELP__73__) in the N-terminal lumen loop (Supplemental Figure 1). The loss of these amino acids leads to the absence of the signature motif required for the glycosylation modification in N66. (N-X-I/T, Figure 1A). This mutation has been reported in multiple clinical studies (23-25). Patients who carry a homozygous cystinosin(7Δ) mutation develop juvenile/intermediate cystinosis, while those who have one allele of cystinosin(7Δ) together with one allele of a 57 kb genomic DNA deletion at *CTNS* locus — the most common cystinosis mutation that leads to a complete loss of the protein — develop infantile/severe cystinosis. Previously, this mutant was reported as a glycosylation-defective variant of cystinosin with a short half life. It was also suggested that 7Δ is degraded at the lysosome, either through ER-phagy or an unknown mechanism that directly transports 7Δ from the ER to the lysosome, bypassing the Golgi (20).

Consistent with the previous study, the full-length 7Δ-GFP mutant runs at a lower molecular weight (approximately 30 kDa smaller) than WT cystinosin-GFP and degrades quickly in a CHX chase (band 1, Figure 1, B and C). Besides the full-length protein, we also observed that cystinosin(7Δ)-GFP has 4 prominent smaller bands, which we termed bands 2 to 5 (Figure 1B). Interestingly, band 3 was also quickly degraded, whereas bands 2, 4, and 5 were relatively stable over time. Bands 2 to 5 were also detected for WT cystinosin-GFP, although their intensities were markedly reduced (Figure 1B).

Besides immunoblotting, we verified the degradation of cystinosin(7Δ)-GFP via flow cytometry, where we observed a major reduction in GFP fluorescence after 24 hours of CHX treatment (Figure 1D). Using an endogenous cystinosin antibody, we verified that the untagged cystinosin(7Δ) also has a similar band pattern and is degraded in a CHX chase (Figure 1, E and F). Finally, we repeated the CHX chase experiment using fibroblasts from a patient homozygously encoding cystinosin(7Δ). We observed a similar pattern, with the full-length 7Δ gradually disappearing over time (Figure 1G). Intriguingly, the endogenous antibody also revealed a small population of higher molecular weight species at approximately 75 kDa (Figure 1, E–G), which will be further discussed below.

### Cystinosin(7Δ)-GFP localizes to both the ER and lysosomes.

**Figure 2:**
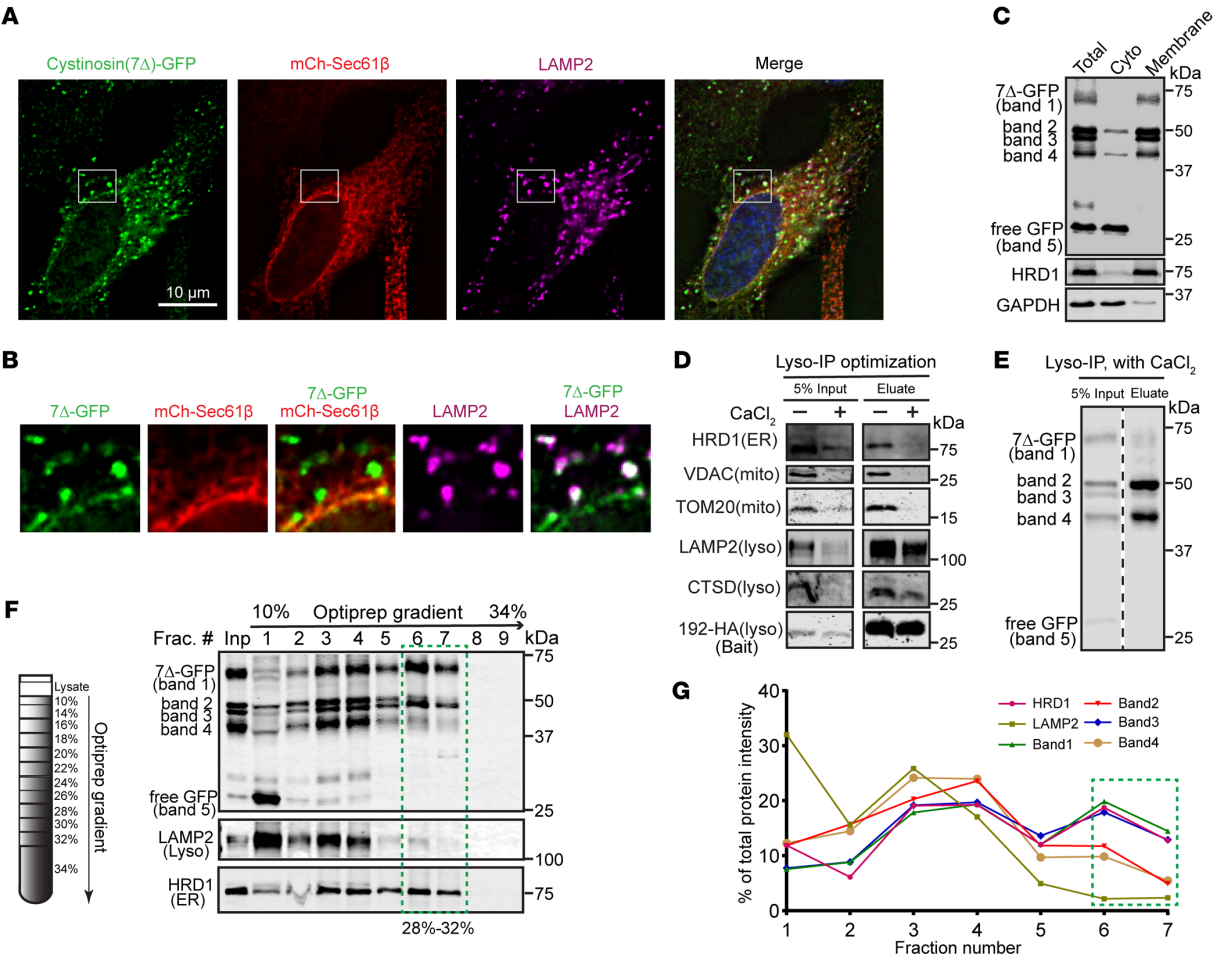
Subcellular localization of cystinosin(7Δ)-GFP. (**A**) Fluorescence microscopy showing the localization of cystinosin(7Δ)-GFP in HeLa cells. 7Δ-GFP is shown in green, ER is labeled in red (mCh-Sec61β), and lysosomes are labeled in magenta (immunostained against LAMP2). Scale bar: 10 μm. (**B**) Enlargement of boxed region in **A**. Red and green overlay is shown in yellow, while magenta and green overlay is shown in white. (**C**) Cytosol and membrane fractions of cystinosin(7Δ)-GFP. (**D**) The ER and mitochondria contamination in a conventional lyso-IP experiment can be cleared out by pretreating cell lysates with 8 mM CaCl_2_. (**E**) Bands 2 and 4 are enriched in the lysosome fraction. (**F**) A representative optiprep density gradient fractionation demonstrating bands 1 and 3 are enriched in fractions 6 and 7 that contain ER (labeled with HRD1) but not lysosome (labeled with LAMP2). The experiment was replicated 3 times with consistent results. (**G**) Quantification of figure (**F**).

To understand the turnover mechanism of cystinosin(7Δ)-GFP, we first needed to identify its cellular localization. In HeLa cells stably expressing cystinosin(7Δ)-GFP, we transiently overexpressed mCherry-Sec61b to label the ER. We also labeled the lysosomes by immunostaining for LAMPp2. Interestingly, cystinosin(7Δ)-GFP had a dual localization to ER and lysosomes (Figure 2, A and B). To understand the localization further, we performed biochemical fractionations. First, we used ultracentrifugation to separate the membrane and the cytosolic components and verified that bands 1 to 4 of cystinosin(7Δ)-GFP are predominantly membrane-bound, while band 5 is cytosolic (Figure 2C). This suggests that band 5 is likely a cytoplasmic cleavage product of the GFP fusion. In order to understand which bands localize to the lysosome, we immunoprecipitated intact lysosomes using the well-established Lyso-IP method (26). In our initial attempts, we noticed substantial ER and mitochondrial contamination in the precipitates (Figure 2D, third lane). Therefore, we pretreated cell lysates with 8 mM CaCl__2__ (see methods for details) to clear out the ER and mitochondria contaminants and repeated the Lyso-IP experiment (Figure 2D, fourth lane). With the improved Lyso-IP method, we observed the enrichment of bands 2 and 4 and negligible amounts of bands 1 and 3 (Figure 2E). We then performed a converse experiment wherein we used an optiprep density gradient to isolate ER from lysosomal fractions (27). After ultracentrifugation, we observed that fractions 6 and 7 gave lysosome-depleted ER fractions in which bands 1 and 3, but not 2 and 4, were enriched (Figure 2, F and G, boxed area). Taken together, we concluded that bands 1 and 3 are the ER-localized population, and bands 2 and 4 are the lysosome-localized population of cystinosin(7Δ)-GFP.

### The 30kDa size difference between cystinosin(7Δ)-GFP and cystinosin-GFP is due to post-ER glycosylation.

After identifying that the full-length (band 1) cystinosin(7Δ)-GFP is retained in the ER, we asked if the large molecular weight difference between band 1 (approximately 70 kDa) and WT cystinosin-GFP((approximately 100 kDa) was due to the lack of Golgi modifications on the N-linked glycans, instead of the mutation that affects the N66 glycosylation. In support of this assumption, the N66A mutant does not migrate at the same size as cystinosin(7Δ)-GFP (Supplemental Figure 2, A and B), even though the mutation slightly reduces the protein size (approximately 90 kDa). We also mutated the other 6 putative N-linked glycosylation sites, but none of the N-to-A mutations reduced the protein size to the cystinosin(7Δ)-GFP range (Supplemental Figure 2B).

**Figure 3:**
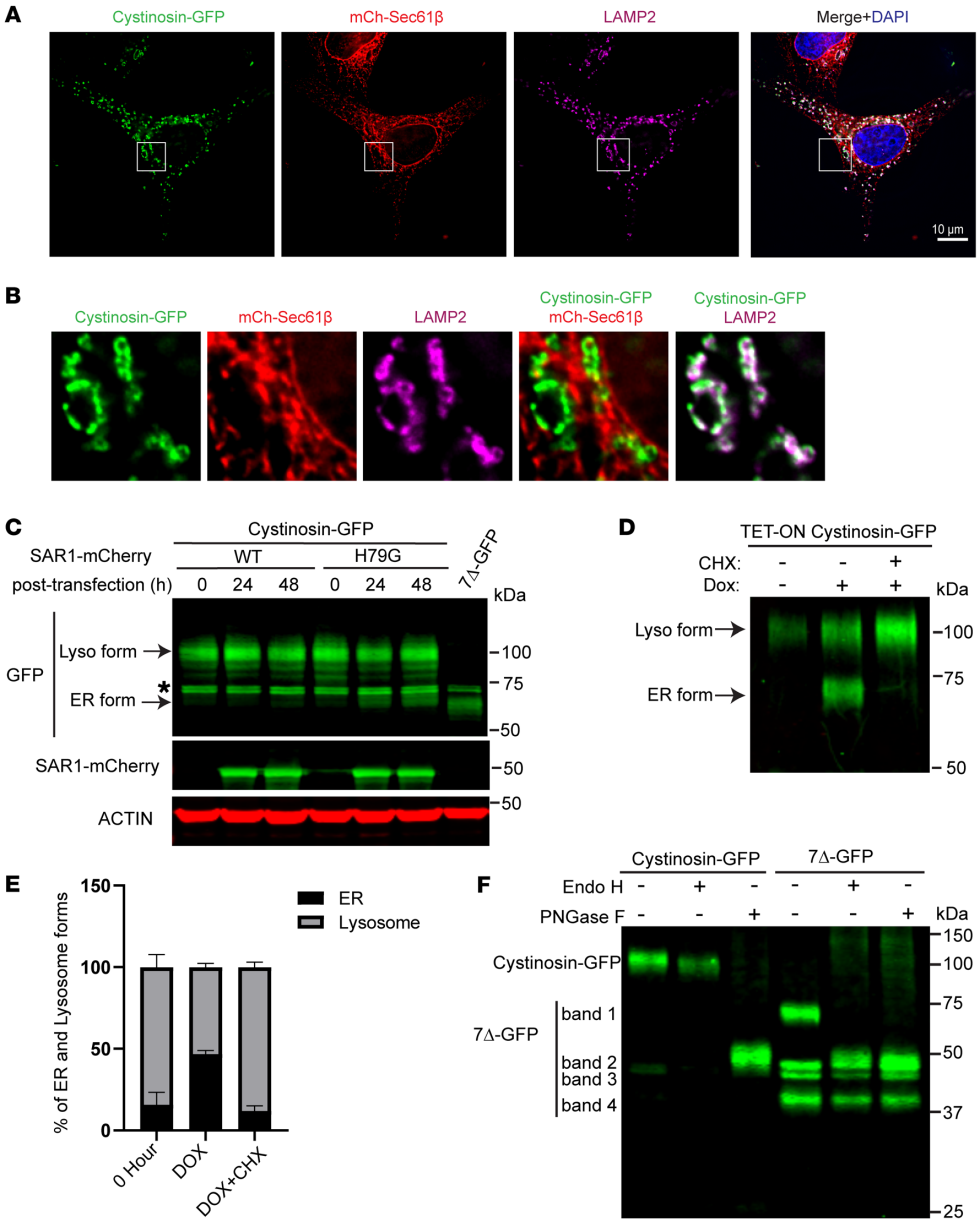
The ER and lysosome forms of cystinosin-GFP. (**A**) Fluorescence microscopy showing the localization of WT cystinosin-GFP in HeLa cells. Cystinosin-GFP is shown in green, ER is labeled in red (mCh-Sec61β), and lysosomes are labeled in magenta (immunostained against LAMP2). Scale bar: 10 μm. (**B**) Enlargement of boxed region in **A**. Red and green overlay is shown in yellow, while magenta and green overlay is shown in white. (**C**) Overexpression of dominant-negative Sar1(H79G)-mCherry leads to the accumulation of an approximately 75 Kda band for WT Cystinosin-GFP, similar to the ER form of 7Δ-GFP. The asterisk highlights a nonspecific band. (**D**) Newly induced WT Cystinosin-GFP has a prominent 75 kDa ER form before it matures into the 100 kDa lysosome form. (**E**) Quantification of ER and lysosome forms in **D**. Error bars: SD calculated from 3 independent replicates. (**F**) Endo H and PNGase F treatment of Cystinosin-GFP and cystinosin(7Δ)-GFP.

We then tested the hypothesis that 2 forms of cystinosin might exist during its maturation (i.e., a smaller ER form and a much larger post-Golgi form) by studying the trafficking of WT cystinosin-GFP. Unlike cystinosin(7Δ)-GFP, we observed an almost complete lysosomal localization for WT cystinosin-GFP (Figure 3, A and B). Consistent with the imaging results, most WT cystinosin-GFP migrates at 100 kDa (Figure 3C, 0 hr). We then overexpressed the dominant negative form (H79G) of SAR1A, a GTPase important in forming COP II vesicles (28). By expressing the dominant negative version of Sar1, cargoes can be retained in the ER without proceeding further into the secretory pathway. Under this condition, we observed an accumulation of an approximately 75 kDa protein population, similar to the size of cystinosin(7Δ)-GFP. In contrast, overexpression of WT SAR1A did not lead to the accumulation of the 75 kDa band (Figure 3C). The approximately 5 kDa difference might be due to the 7 AA deletion in 7Δ.

As an alternative method to confirm the existence of ER and lysosome forms, we expressed WT cystinosin-GFP under a TET-ON promoter. Without induction, only a small amount of cystinosin-GFP was produced due to the leaky expression of the TET-ON promoter. Upon doxycycline induction, we observed 2 major bands on the immunoblot, which corresponded to the ER (approximately 75 KDa) and the lysosome form (approximately 100 KDa). After adding CHX for 3 hours, the ER form was largely reduced while the lysosome form continued to increase (Figure 3, D and E), suggesting that the newly synthesized cystinosin-GFP was trafficked from ER to the lysosome.

Finally, to verify that the 70 kDa cystinosin(7Δ)-GFP bands represent the incompletely glycosylated high-mannose ER form, we performed deglycosylation assays using Endo H and PNGase F. Endo H is an enzyme specifically targeting mannose-rich N-linked glycans, while PNGase F is capable of cleaving all forms of N-linked glycans. In the Golgi apparatus, N-linked glycans undergo a series of modifications. Initially, α-mannosidase II removes 2 mannose residues from the N-linked glycans. Subsequently, complex sugar structures, such as sialic acid, galactose, and N-acetylgalactosamine, are added. These modifications lead to the formation of complex glycans, which are resistant to Endo H treatment. As shown in Figure 3F, we observed that WT cystinosin-GFP is only sensitive to PNGase F. At the same time, cystinosin(7Δ)-GFP is sensitive to both Endo H and PNGase F, supporting that 7Δ has not obtained the Golgi-based glycosylation modifications.

Importantly, the sizes of bands 2–4 did not change after PNGaseF treatment, indicating that they do not have any N-linked glycosylation. Furthermore, the deglycosylated cystinosin(7Δ)-GFP has a similar size to band 2, suggesting that band 2 might represent a nonglycosylated full-length cystinosin(7Δ)-GFP that somehow reaches the lysosome. This result demonstrates that glycosylation is not necessary for the transport of cystinosin out of the ER. We conducted an experiment where we substituted all 7 asparagine residues in the cystinosin lumen (i.e., the putative N-linked glycosylation sites) with alanine, creating the 7NA mutant. Remarkably, this mutant variant, despite lacking glycosylation, still effectively reached the lysosome, as evidenced by the lyso-IP and colocalization findings depicted in Supplemental Figure 2, C and D. The targeting of cystinosin to the lysosome relies on 2 key elements: the C-terminal GYDQL motif and the second PQ motif (17). Notably, when these motifs were deleted, the 7NA-GFP construct became trapped within the ER, as observed in Supplemental Figure 2D.

Taken together, we concluded that band 1 represents a partially glycosylated form because most of the protein cannot traffic out of the ER. If the protein successfully exits the ER, it will be further modified at the Golgi. The lysosome form should be approximately 100 kDa for cystinosin-GFP and approximately 75 kDa for nontagged cystinosin. Interestingly, with the endogenous antibody, we did observe a small fraction of approximately 70 kDa band in patient fibroblasts (Figure 1G), suggesting some cystinosin(7Δ) can reach the lysosome. This could explain the juvenile/intermediate phenotype observed in homozygous patients. The lysosome form of cystinosin(7Δ)-GFP is barely detectable with the GFP antibody, probably due to the sensitivity difference between the 2 antibodies. However, after enriching cystinosin(7Δ)-GFP with immunoprecipitation, we did observe a small population of lysosome forms even with the GFP antibody (Supplemental Figure 2E, white box).

### Band 3 of cystinosin(7Δ) was due to the internal translation initiated at Met^148^.

Besides band 2, bands 3 and 4 also lack glycosylation modifications but can still be recognized by the antibody that recognizes the C-terminal GFP. These results suggest that bands 3 and 4 are N-terminal truncation products that derive from either an internal translation initiation or breakage during — or after — the protein synthesis. There are 4 internal methionines in the cystinosin coding sequence (148, 252, 287, and 316, Supplemental Figure 3A). To test the possibility of internal translation initiation, we generated N-terminal truncation constructs that start with Met^^148^^, Met^^252^^, Met^^287^^, and Met^^316^^. When expressed, M148-GFP generated a band the same size as band 3, while the rest were expressed as truncation products even smaller than band 4 (Supplemental Figure 3B). Furthermore, band 3 was abolished when Met^^148^^ was mutated to Ala within cystinosin(7Δ)-GFP(Supplemental Figure 3C). These results suggested that band 3 is created by an internal translation initiation that starts at Met^^148^^, whereas band 4 is likely created by a protein breakage between Met^^148^^ and Met^^252^^.

### Cystinosin(7Δ)-GFP is degraded at the proteasome, not the lysosome.

With the understanding of the localization of the different populations of cystinosin(7Δ)-GFP, we proceeded to investigate the degradation mechanisms involved. As shown in Figure 2, the two fast-degrading populations, bands 1 and 3, are both localized to the ER. ER-localized proteins are either degraded via the proteasome through the ERAD pathway or degraded at the lysosome through ER-phagy or ER-to-lysosome vesicular trafficking (29).

**Figure 4:**
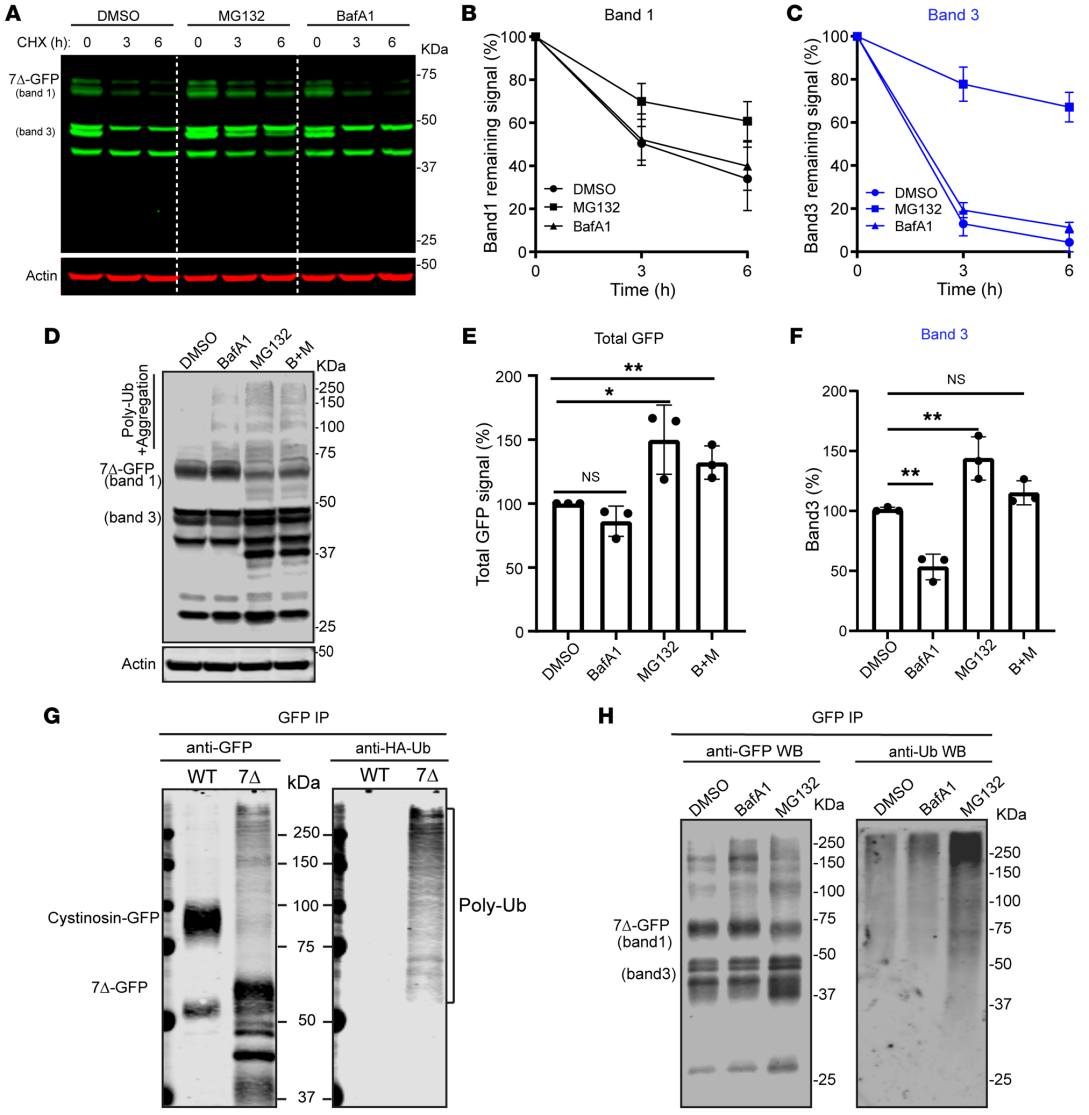
Cystinosin(7Δ)-GFP is degraded by the ubiquitin-proteasome pathway. (**A**) CHX chase assay of cystinosin(7Δ)-GFP in cells treated with either DMSO, BafA1 (400 nM) or MG132 (10 μM). (**B** and **C**) Quantification of bands 1 and 3 in **A**. (**D**) Steady-state protein levels of cystinosin(7Δ)-GFP after DMSO, BafA1 (400 nM), MG132 (10 μM), or a combination of both BafA1 and MG132 treatments. (**E** and **F**) Quantification of relative protein levels of either the whole lane (**E**) or band3 **(F**) in **D**. The statistical analysis was conducted with the ordinary 1-way ANOVA. (**G**) Cystinosin(7Δ)-GFP, but not WT cystinosin-GFP, is polyubiquitinated. (**H**) The polyubiquitination of cystinosin(Δ7)-GFP is increased after MG132 but not BafA1 treatment. Error bars: SD. All quantifications and data analysis were performed based on 3 independent replicates. *P ≤ 0.05, **P ≤ 0.01.

To determine the site of degradation, we treated cells expressing stable cystinosin(7Δ)-GFP with either Bafilomycin A1 (an inhibitor of lysosome-mediated degradation) or MG132 (an inhibitor of proteasome-mediated degradation). The results of these drug treatments revealed that the degradation of both bands 1 and 3 was partially inhibited by MG132, while BafA1 had a minimal effect on degradation (Figure 4, A–C). Additionally, we were interested in examining the changes in steady-state protein levels. As illustrated in Figures 4, D–F, 6 hours of treatment with MG132 clearly stabilized band 3. Furthermore, we did not observe a distinct increase in band 1; instead, we observed a ladder-like pattern reminiscent of polyubiquitination or membrane protein aggregation (30). Moreover, we detected additional bands between 50 and 75 kDa, as well as a band at approximately 37 kDa, which could represent degradation intermediates of Band 1 and Band 3 that were stabilized by MG132. The intensity of the entire lane increased by about 1.5-fold for MG132-treated samples (Figure 4E). It is worth noting that similar stabilization of other ERAD substrates by MG132 has been reported previously (31, 32). In contrast, BafA1 only induced a minor accumulation of higher molecular weight species compared with the DMSO control, likely due to an indirect effect caused by autophagy inhibition (33, 34).

To further characterize the higher molecule species formed after MG132 treatment, we immunoprecipitated the protein from cells and probed for ubiquitin. Without any treatment, a fraction of the cystinosin(7Δ)-GFP was already polyubiquitinated, consistent with the hypothesis that the mutant is constantly being ubiquitinated and degraded (Figure 4G). Subsequent treatment with MG132, but not with BafA1, resulted in enhanced polyubiquitination (Figure 4H). Interestingly, when we treated the samples with the deubiquitinase USP7, the higher molecule species were only partially reduced, indicating the presence of a mixture of both polyubiquitinated populations and membrane protein aggregates (Supplemental Figure 3). This observation suggests that cystinosin(7Δ)-GFP has a propensity to aggregate, particularly when it cannot be eliminated through proteasome-mediated degradation under MG132 treatment. Collectively, we concluded that both bands 1 and 3 of cystinosin(7Δ)-GFP are degraded in a proteasome-dependent manner. Lysosomes are not involved in their degradation process.

### Cystinosin(7Δ)-GFP is degraded via the ERAD pathway.

**Figure 5:**
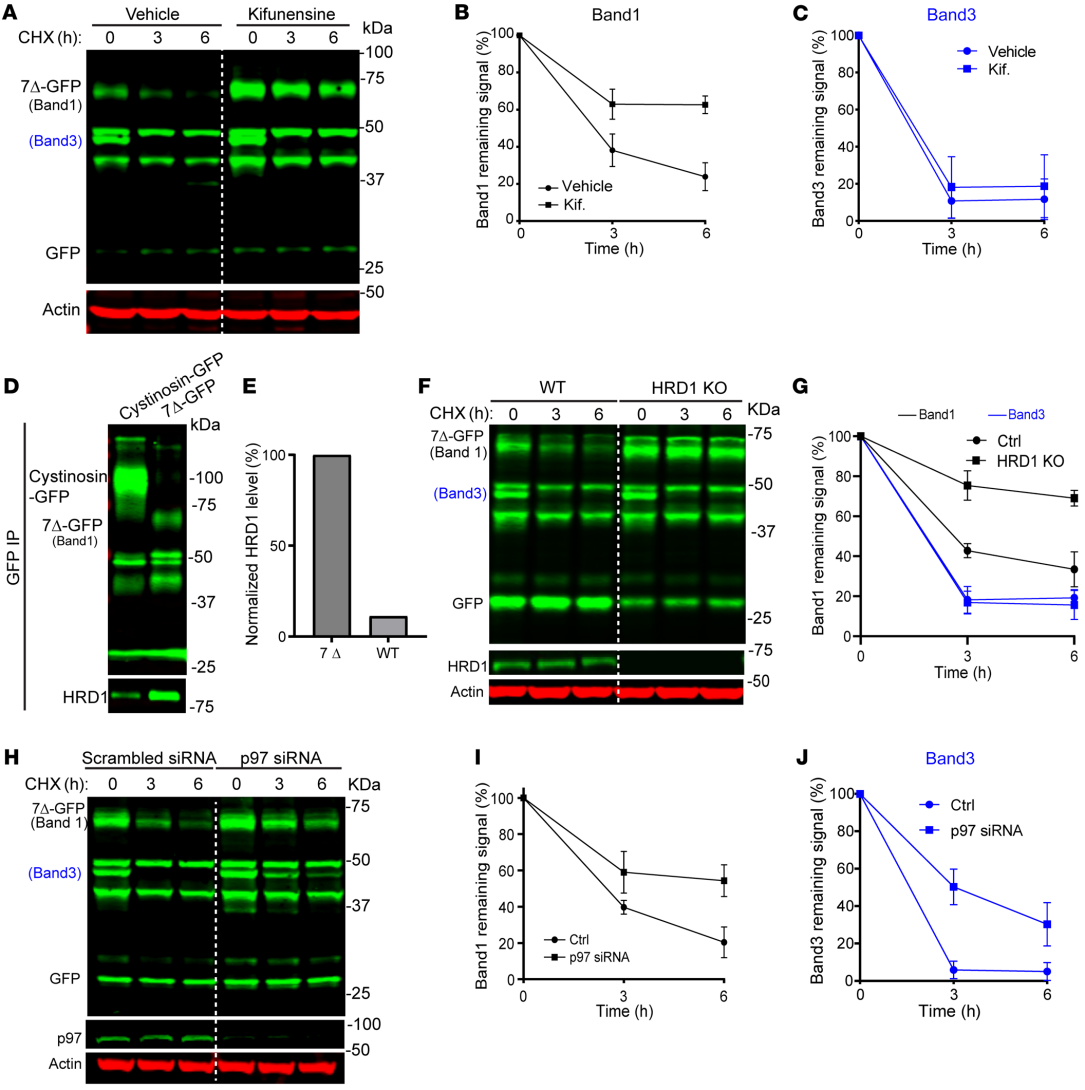
Cystinosin(7Δ)-GFP is degraded by the ERAD pathway. (**A**) CHX chase assay of cystinosin(7Δ)-GFP in cells pretreated with either vehicle or Kifunensine (1.5 μg/mL). Kifunensine was treated for 16 hours before addition of CHX. (**B** and **C**) Quantification of bands 1 and 3 in **A**. (**D**) Cystinosin(7Δ)-GFP has a stronger interaction with HRD1 than WT cystinosin-GFP. (**E**) Relative levels of HRD1 precipitated by either cystinosin(7Δ)-GFP (100%) or WT cystinosin-GFP (1.12%). (**F**) Knocking out HRD1 stabilizes band 1, but not band 3, of cystinosin(7Δ)-GFP. (**G**) Quantification of relative protein levels of bands 1 and 3 in **E**. (**H**) Knockdown stabilizes both bands 1and 3 of cystinosin(7Δ)-GFP. (**I** and **J**) Quantification of relative protein levels of bands 1and 3 in **G**. Error bars: SD. All quantifications and data analysis were performed based on 3 independent replicates.

After identifying that cystinosin(7Δ)-GFP is degraded in the ER using the ubiquitin-proteasome machinery, we next asked if this degradation was through the ERAD pathway. ERAD is a well-characterized mechanism for the removal and degradation of unnecessary or misfolded ER proteins (35, 36). It begins with the recognition of misfolded proteins, often involving N-linked glycans for glycoproteins. Within the ER, the initial high-mannose glycan (Glc3Man9GlcNAc2) undergoes sequential trimming by glucosidase I and II, resulting in the formation of the glycan structure GlcMan9GlcNAc2. This glycan structure is recognized by the chaperones calnexin and calreticulin (CANX/CALR), which assist in the folding of the polypeptide into its native conformation. The folding process facilitated by CANX/CALR concludes when the final Glc residue is removed by glucosidase II, liberating the glycoprotein. Properly folded proteins proceed to exit the ER, while misfolded ones are recognized by UDP glucose:glycosyltransferase (UGGT), leading to their reglucosylation and return to the CANX/CALR cycle. To prevent proteins from entering an infinite loop in the CANX/CALR cycle, terminally misfolded glycoproteins are identified by ER degradation-enhancing α-mannosidase-like lectins (EDEMs) and targeted for degradation (37, 38). To test if EDEMs played a role in the degradation of cystinosin(7Δ)-GFP, we treated cells with Kifunensine, a mannosidase inhibitor often shown to inhibit EDEM-dependent degradation of proteins (39). Indeed, treating cells with kifunensine stabilized the degradation of full-length cystinosin(7Δ)-GFP (Figure 5, A–C) and increased its steady-state levels. Importantly, the nonglycosylated band 3 was not stabilized by Kifunensine treatment, underscoring the importance of glycosylation in EDEM-dependent ER protein degradation.

If proteins fail to fold properly at the ER, they are ubiquitinated by E3 ligases. HRD1 is the most prevalent ER-localized E3 ligase involved in ERAD (40). It also contributes to the formation of the retrotranslocation channel to shuttle ERAD substrates out of the ER. To test the role of HRD1 in the degradation of cystinosin(7Δ)-GFP, we immunoprecipitated the mutant and confirmed that it interacted readily with HRD1. The interaction is much stronger than the WT cystinosin-GFP (Figure 5, D and E). We further knocked out HRD1 using the CRISPR-Cas9 technology, which stabilized the degradation of cystinosin(7Δ)-GFP, confirming the importance of this E3 ligase in the turnover mechanism. Interestingly, band 3 degradation was not stabilized by HRD1 KO, indicating that a different E3 ligase might be responsible for ubiquitinating band 3 (Figure 5, F and G).

Once ubiquitinated, membrane substrates are extracted by the p97-Ufd1-Npl4 complex (41). The p97-UFD1-NPL4 complex hydrolyzes ATP to pull the ubiquitinated polypeptide out of the ER membrane before delivering it to the proteasome for proteolysis. To confirm the importance of p97, we knocked down its protein level using siRNA. We observed stabilization of both bands 1 and 3 of cystinosin(7Δ)-GFP, indicating that p97 was critical for the degradation of both ER forms (Figure 5, H–J).

In summary, we conclude that cystinosin(7Δ)-GFP is recognized by the ER lumenal chaperones, ubiquitinated by the ER membrane E3 ligase HRD1, and extracted by the cytosolic p97 ATPase before its degradation at the proteasome, hence following every step of the well-established ERAD pathway. Interestingly, through IP and mass spectrometry, Nevo et al. demonstrated that cystinosin(7Δ) interacts with several ER quality control machinery, such as calnexin, OS9, and Sel1L, which is consistent with our conclusion that cystinosin(7Δ) is degraded by the ERAD pathway (20).

### Live-cell imaging verifies the degradation of 7Δ-GFP at the ER.

**Figure 6:**
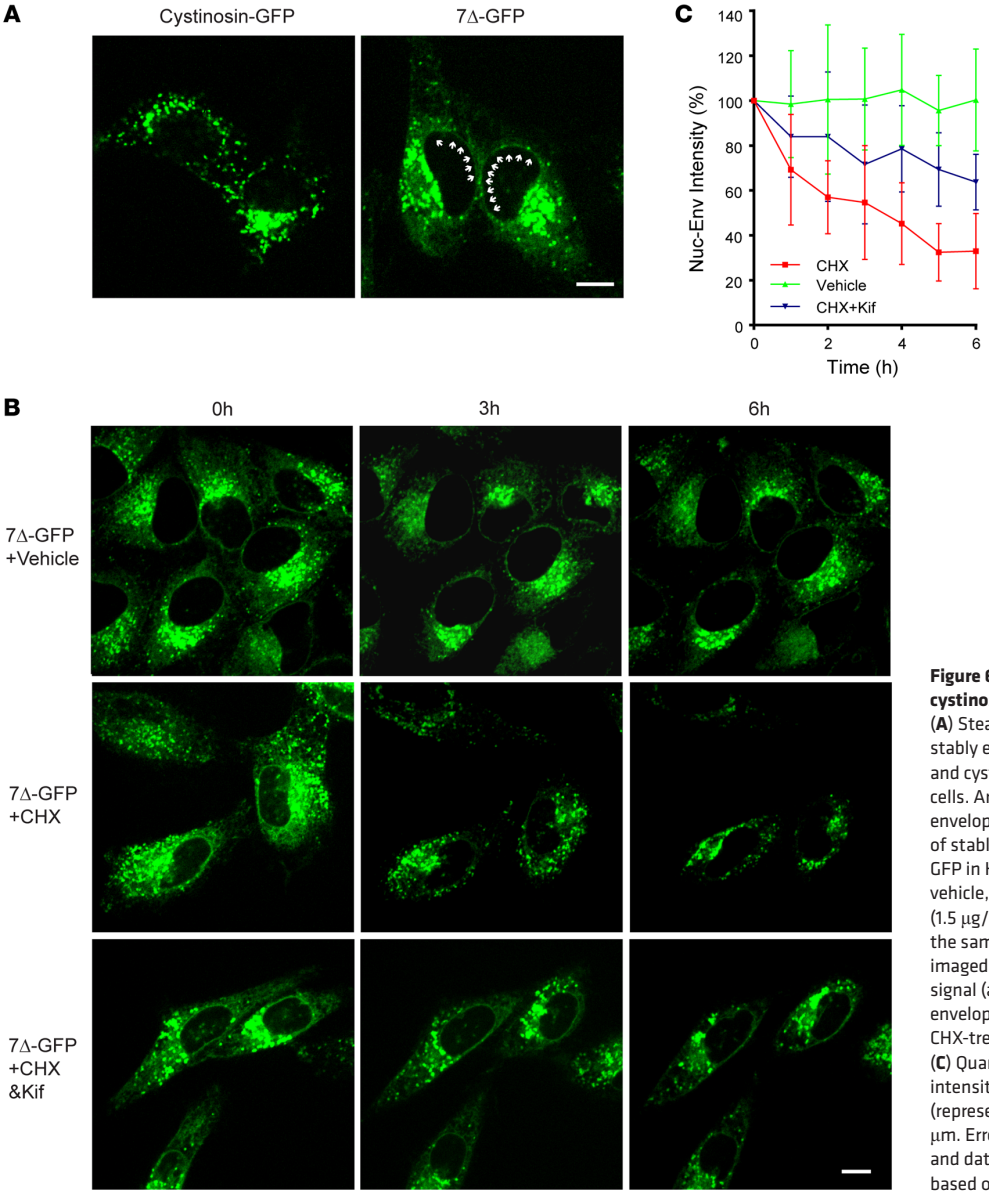
Live-cell imaging of cystinosin(7Δ)-GFP degradation. (**A**) Steady-state localization of stably expressed WT cystinosin-GFP and cystinosin(7Δ)-GFP in live Hela cells. Arrows highlight the nuclear envelope signal. (**B**) Live-cell imaging of stably expressed cystinosin(7Δ)-GFP in Hela cells treated with either vehicle, CHX, or CHX with kifunensine (1.5 μg/mL). For each treatment, the same cells were continuously imaged for 6 hours. Note that the ER signal (as indicated by the nuclear envelope signal) only disappeared in CHX-treated cells (middle images). (**C**) Quantification of fluorescent intensity of nuclear envelope signal (representing ER) in **B**. Scale bar: 10 μm. Error bars: SD. All quantifications and data analysis were performed based on 3 independent replicates.

Next, we performed live-cell imaging to visualize the degradation of cystinosin(7Δ)-GFP. At steady state, we observed that WT cystinosin-GFP was entirely lysosomal (puncta) while cystinosin(7Δ)-GFP had both lysosome (puncta) and ER (fiber-like and nuclear envelope) signals (Figure 6A, Supplemental Videos 1 and 2). After adding cycloheximide, most of the ER signal was lost, as demonstrated by the loss of nuclear envelope signals, whereas the lysosome punctae remained constant throughout the time course (Supplemental Video 3 and Figure 6, B and C). In contrast, the vehicle control had a negligible loss of ER signal (Figure 6, B and C, and Supplemental Video 4). Finally, we observed that the addition of Kifunensine stabilized the ER signal in the CHX chase experiment, in correspondence with what we observed in Figure 4A (Figure 6, B and C, and Supplemental Video 5). These imaging results independently confirmed that the degradation of cystinosin(7Δ)-GFP occurs in the ER

### Chemical chaperones can assist in the folding and ER-exit of cystinosin(7Δ).

**Figure 7:**
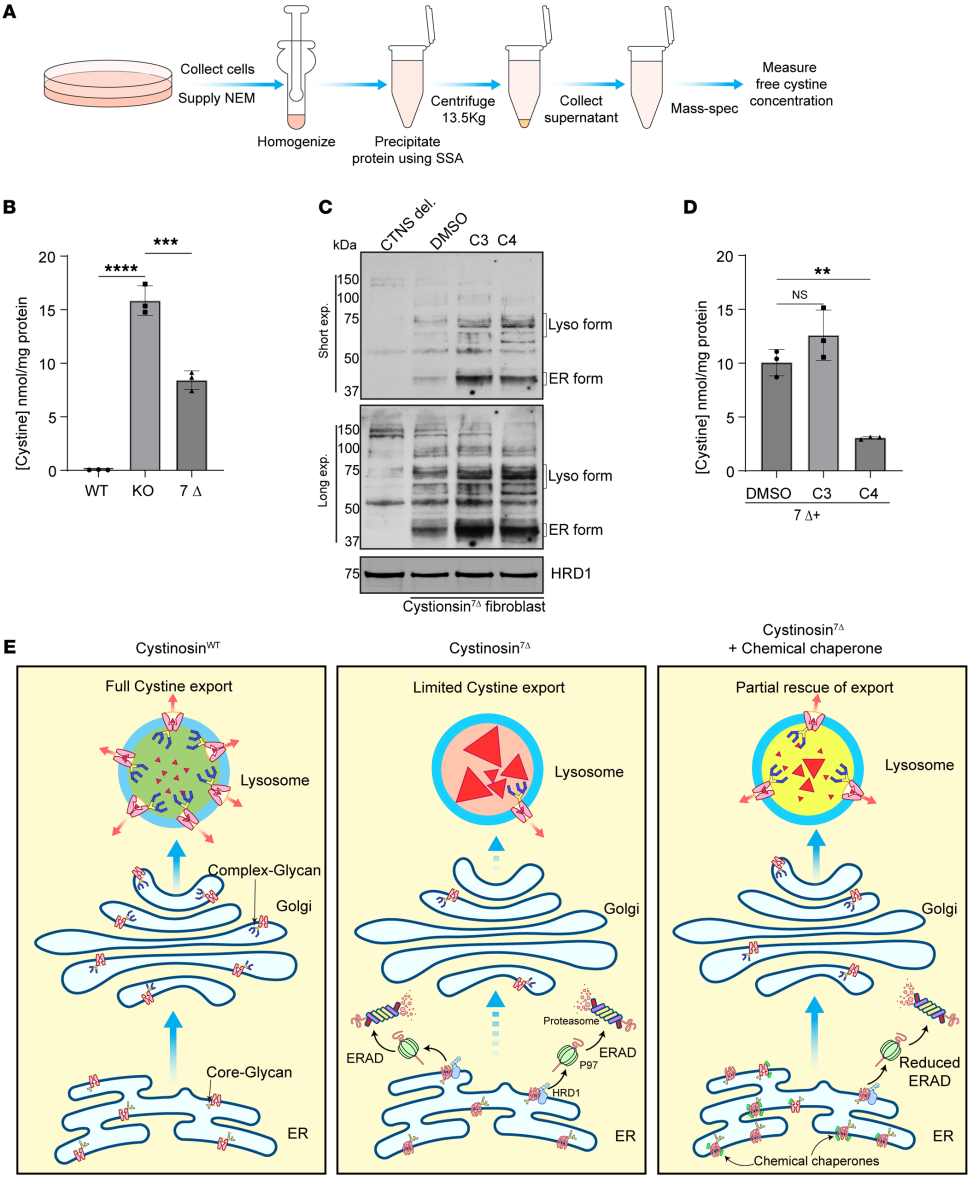
Chemical chaperones can restore the stability and functionality of cystinosin(7Δ)-GFP. (**A**) Schematic representation of the protocol employed to analyze lysosomal cystine levels. (**B**) Lysosomal cystine levels in healthy (WT), 57kb genomic DNA deletion (KO), or 7Δ cystinotic fibroblasts. (**C**) Treatments with chemical chaperones stabilized both ER and lysosome forms of endogenous cystinosin(7Δ). (**D**) Lysosomal cystine levels in 7Δ cystinotic fibroblasts when treated with chemical chaperones. (**E**) A model to depict the pathogenesis of cystinosin(7Δ) and the effects of chemical chaperones. Error bars: SD. The statistical analysis was conducted with the ordinary 1-way ANOVA. All quantifications and data analysis were performed based on 3 independent replicates. **P ≤ 0.01, ****P ≤ 0.0001.

After establishing that cystinosin(7Δ) is degraded through the ERAD pathway, we asked if we could use chemical chaperones or inhibitors of ERAD to help with the folding and trafficking of cystinosin(7Δ) and ultimately ameliorate the disease. Previous studies reported that cystine levels in the white blood cells of healthy individuals are under 0.2 nmol cystine/mg of protein. In heterozygous carriers, the cystine levels range from 0.2 to 1 nmol/mg of protein. For cystinosis patients, the accumulation is within a range of 3 to 20 nmol cystine/mg of protein (15). We first quantified the cystine levels in fibroblasts from individuals with WT cystinosin, with cystinosin(7Δ), and those with the 57kb deletion following a protocol (42) depicted in Figure 7A. Both cystinotic fibroblasts accumulated the cystine levels to the pathogenic range (Figure 7B). Furthermore, the cystine level in cystinosin(7Δ) fibroblasts was significantly lower than that of the 57 kb deletion (8.42 versus 15.85 nmol/mg of protein), possibly due to the existence of a small population of the lysosome form in 7Δ cells (Figure 1G).

As stated above, the most common cystic fibrosis mutant, CFTR ΔF508, is also degraded by the ERAD pathway. Over the years, several chemical chaperones (43) have been developed to prevent the degradation of CFTR ΔF508 and facilitate its exit from the ER (44). Here, we demonstrated that cystinosin(7Δ) is also degraded by the ERAD pathway. We asked if we could employ some of the available CFTR correctors to assist with the folding and ER exit of cystinosin(7Δ). This is an exciting avenue as it not only brings in the concept of precision medicine for treating cystinosis, but also repurposes well-characterized drugs for another disease: cystic fibrosis. We tested 2 prominent CFTR correctors, C3 (VRT-325) and C4(Corr 4a)- (45), and observed that endogenous cystinosin(7Δ) did indeed increase stability of the ER form as well as a portion of the lysosome form (Figure 7C). A similar stabilization has been made for CFTR ΔF508 using these correctors (46). To test if the stabilized lysosome form of cystinosin(7Δ) was functional, we measured lysosomal cystine levels in patient fibroblasts after treating them with the correctors. As shown in Figure 7D, C4 treatment reduced the cystine level by 70% (from 10.06 nmol/mg of protein to 3.06 nmol/mg of protein). Surprisingly, although C3 treatment also stabilized the ER and lysosome forms of cystinosin(7Δ) at the protein level, it did not reduce the cystine level in patient fibroblasts. The reason for this discrepancy remains unclear. Nevertheless, our results demonstrated that cystinosin(7Δ) stability and function could be rescued by repurposing some of the established chemical chaperones to treat CFTR ΔF508.

## Discussion

This study established the role of the ERAD pathway in the pathogenesis of cystinosis for certain cystinosin mutants. Our results indicate that the cystinosin(7Δ) protein product has multiple subpopulations. These include the partially glycosylated full-length protein (band 1), a protein product translated from an internal methionine (band 3), and 2 bands that are likely the nonglycosylated full-length protein (band 2) and an N-terminal truncation product (band 4). While the full-length and internally translated proteins localize to the ER, the nonglycosylated and truncated forms can traffic to the lysosome. Trapping the full-length protein in the ER is also the reason for the 25 kDa size difference between 7Δ and WT because it cannot obtain the complex glycan modification at the Golgi. Importantly, it is the ER forms that degrade rapidly, whereas the lysosomal forms are stable. A similar degradation pattern was also observed for endogenous 7Δ using patient fibroblasts. Furthermore, we identified that the mutant is degraded via the ERAD pathway and can be stabilized by both genetic and chemical inhibition of the individual components of ERAD, including HRD1, p97, proteasome, and EDEMs.

Besides the 4 major bands we analyzed, there is a small population of 7Δ that can reach the lysosome and receive complex glycans at the Golgi. With the GFP antibody, the fully glycosylated lysosome form of 7Δ-GFP can only be detected after we enriched the protein by immunoprecipitation (Supplemental Figure 2D). However, with an endogenous antibody, the lysosome form is easier to detect in patient fibroblasts (Figure 1G). The small amount of fully glycosylated lysosomal 7Δ likely accounts for the juvenile/intermediate phenotypes in homozygous patients, as well as the relatively low accumulation of cystine levels in our measurement (Figure 7B). Interestingly, Gasnier and colleagues measured the transporter activity of 7Δ to be 19% of the WT control, supporting a model where 7Δ, if properly targeted, could export some lumenal cystine (47).

ERAD has been a cellular pathway that has served as a double-edged sword. On the one hand, ERAD plays an important role in cellular physiology by assisting protein folding in the ER and removing improperly folded protein aggregates. This is especially important for cells that are actively growing or specialized in secretion. For instance, the ERAD pathway is upregulated during chondrogenesis, which is the formation of cartilage. Because this process requires the secretion of a large amount of extracellular matrix (ECM) proteins, ERAD upregulation is essential to reduce proteotoxicity (48). On the other hand, ERAD is also known to unnecessarily degrade some mutant proteins that are still functional (49), which leads to human diseases. As stated above, the ΔF508 mutant in the CFTR protein is degraded via ERAD. Similar to cystinosin(7Δ), ΔF508 mutant is still partially functional if it traffics to the plasma membrane (50, 51).

The use of chemical chaperones has helped rescue the ΔF508 mutant from degradation and further facilitated its trafficking to the plasma membrane. The working mechanisms of these chaperones are being actively investigated. For example, a recent cryo-EM structure reveals that 2 of these correctors work by directly binding to the transmembrane domain of ΔF508 and thermodynamically stabilizing the protein (52). It is speculated that other correctors could function either by directly enhancing the folding of mutants or by inhibiting the ERAD pathway and thus giving the mutants more time to fold.

Strikingly, we show that these correctors are versatile and can assist in the folding of cystinosin(7Δ) as well. We showed that 2 prominent correctors, C3 and C4, can stabilize 7Δ and facilitate its ER exit. However, the lysosomal cystine levels are reduced only in C4 treatment. It is important for chemical chaperones to bind to the mutant proteins to help in the folding. However, chaperones may bind too tightly with the mutants. This could prevent the conformation change of transporters during transportation, which could explain why C3-stabilized 7Δ is less functional than the C4-stabilized population. Nevertheless, our work suggests that CFTR correctors can be repurposed (53) to treat cystinosis and possibly other ERAD-dependent diseases (Figure 7E).

Our work emphasizes the importance of understanding the disease mechanism of individual mutants to help establish precision medicine (54, 55). Different patient mutations in the same gene may have their unique pathogenesis and, hence, may require a very different therapeutic strategy.

## Methods

### Mammalian cell culture.

HEK293 (CRL-1573), HEK293T (CRL-3216), and HeLa (CCL-2) cells were obtained from ATCC. The nephropathic cystinosis fibroblasts (GM00706) and the healthy individual fibroblasts (GM05658) were obtained from Coriell Institute for Medical Research. Cystinosin(7Δ) fibroblasts were obtained as a gift from William Gahl, the National Human Genome Research Institute (NHGRI; Bethesda, Maryland, USA)**.** Cell lines were cultured in DMEM (Invitrogen) with 1% penicillin and streptomycin (Invitrogen), 10% super calf serum and 1 μg/ml plasmocin (Invivogen). Primary cells were cultured in Ham’s F12 (Kaighn’s modification) with 1% penicillin and streptomycin, 10% FBS and 1 μg/ml plasmocin. All cells were cultured at 37°C, 5% CO__2,__ and tested negative for mycoplasma contamination using the Mycoalert mycoplasma detection kit (Lonza). Details of the cell lines used in this study can be found in Supplemental Table 1.

### Plasmids.

The CDS of cystinosin was purchased from DNAsu (pANT7-*CTNS*-cGST). The CDS was amplified and cloned into p-EGFPN1 using the sites XhoI and SacII to overexpress cystinosin with a c-terminal GFP tag in HEK293 cells. The cystinosin(7Δ)-GFP was further constructed on the WT clone using overlapping extension PCR. For the generation of cell lines stably expressing either WT or 7Δ cystinosin-GFP, GFP was fused to the C-terminal of cystinosin with 2 × Gly-Gly-Gly-Ser linker using overlapping extension PCR and cloned using NotI and BamHI sites of the mAG-Gal3 plasmid (addgene). This construct is referred to as pHAGE2-*CTNS*(WT/7Δ)-GFP-IRES-PURO (Supplemental Table 3). Various lentivirus plasmids with different selection markers were generated from pHAGE2-*CTNS*-GFP-IRES-PURO by swapping out the puromycin resistance genes using NdeI and ClaI sites and inserting a blasticidin resistance gene or hygromycin resistance gene. pcDNA5-FRT-Flag-Venus-Sec61β (mouse) was a gift from Yusong Guo, The Hong Kong University of Science and Technology (Clear Water Bay, Hong Kong)**.** Using this plasmid, sec61β CDS was amplified and cloned into the sites EcoRI and SalI of the plasmid pmCherry C1, to overexpress mCherry-Sec61β in HeLa cells. The 2 plasmids used for generating stable cell lines for Lyso-IP, pLJC5-TMEM192-2XFLAG, and pLJC5-TMEM192-3XHA, were obtained from addgene. The doxycycline-inducible expression of cystinosin was achieved by cloning the *CTNS* CDS between sites NheI and Age in the plasmid pCW57.1, which was a gift from Jacob Kitzman, University of Michigan. The *CTNS* truncation products starting from internal methionines were generated by PCR amplification using *CTNS* CDS as a template. This PCR product was cloned into pEGFPN1 using the sites XhoI and SacII. The pHAGE2-*CTNS*(7Δ+M148A)-GFP was constructed using site-directed mutagenesis of pHAGE2-*CTNS*(7Δ)-GFP. The individual glycosylation mutants were built using site-directed mutagenesis on pHAGE2-*CTNS*-GFP. Virus packaging plasmids psPAX2 (Addgene 12260) and pMD2.G (Addgene 12259) were purchased from Addgene.

### Transient transfection.

All transfections were performed in HEK293 or HeLa cells that were approximately 70% confluent. 2.4 μg of plasmid DNA was mixed with Lipofectamine 2000 (Invitrogen) per the manufacturer’s instructions and added to cells. The medium was changed 4-to-6 hours after transfection, and cells were harvested for Western blotting or fixed for imaging experiments 24 or 48 hours after transfection.

### Cycloheximide chase.

Cycloheximide (Sigma-Aldrich) was added to a final concentration of 100 μg/mL in HEK293 and HeLa cells, while, in fibroblasts, they were added to a final concentration of 120 μg/mL. Cells were harvested at indicated time points for Western blot analysis.

### siRNA knockdown.

HEK293 cells were transfected with siRNAs using Lipofectamine RNAimax (Invitrogen) according to the manufacturer’s instructions. After 24 hours, cells were transfected with the same amount (100 nM) of siRNA again. 72 hours after the first round of transfection, cells were subjected to CHX chase and harvested for Western blot analysis.

The following siRNA sequences were used: p97, 5′-CCUGAUUGCUCGAGCUGUA-3′ and ON-TARGETplus nontargeting pool (Dharmacon), 5′-UGGUUUACAUGUCGACUAA-3′, 5′-UGGUUUACAUGUUGUGUGA-3′, 5′-UGGUUUACAUGUUUUUCUGA-3′, and 5′-UGGUUUACAUGUUUUCCUA-3′.

### Generation of HRD1 KO cell line using CRISPR-Cas9 technology.

HRD1-KO HEK293 cells were generated as described Ran et al. (56). The knockout was achieved by the sgRNA sequence 5′-CTTGGTCAGGTACACCACAG-3′. The sgRNAs were ligated into pspCas9(BB)-2A-Puro and 2.4 μg of the cloned plasmid was transfected into HEK 293 cells using Lipofectamine 2000 according to the manufacturer’s instruction. 24 hours after transfection, cells were treated with 1 μg/mL puromycin (Invitrogen) for 72 hours to select for cells that contained the plasmid. Single cells were isolated into 96-well plates using limited dilution to a final concentration of 0.5 cells per well. The individual colonies were expanded and screened for HRD1 KO via Western blotting.

### Generation of lentiviral stable cell lines.

The Lentiviral stable cell lines were generated as described by Zhang, et al. (57, 58). HEK293T cells were transfected with transfer plasmid, psPAX2 (Addgene 12260), and pMD2.G (Addgene 12259) at 3.5:3.5:1 ratio using Lipofectamine 2000 according to the manufacturer’s instruction. 3 days after transfection, the virus-containing supernatant was collected using a 5 mL syringe and applied through a 0.45 μm filter. To generate stable cell lines, HEK293, HEK293T, or HeLa cells were seeded in 3.5 cm dishes and infected with the virus-containing supernatant (DMEM containing 10% super calf serum, 10 μg/ml polybrene, MOI between 0.3 to 0.4). For puromycin selection, the media was refreshed with DMEM containing 10% super calf serum and 1 μg/mL puromycin. The selection lasted for at least 3 days before subsequent analysis.

### Protein lysate preparation and Western blot protocol.

Cells were harvested in ice-cold 1 × PBS and centrifuged at 800*g* for 1 minute. Cell pellets were lysed in lysis buffer (20 mM Tris pH 8.0, 150 mM NaCl, 1% Triton) containing 1 × protease inhibitor cocktail (Biotool) at 4°C for 20 minutes. Subsequently, samples were centrifuged at 18,000*g* for 15 minutes at 4°C. Protein concentration was measured using Bradford reagent according to the manufacturer’s instructions, and all samples were normalized to an equal protein concentration. An equal volume of 2 × urea sample buffer (150 mM Tris pH 6.8, 6 M Urea, 6% SDS, 40% glycerol, 100 mM DTT, 0.1% bromophenol blue) was added to the lysates and heated at 65°C for 5 minutes. 20 μg of each lysate was loaded and separated on an 11% (unless otherwise mentioned) SDS-PAGE gel. Protein samples were transferred to a nitrocellulose membrane for Western blot analysis. After incubation with primary and secondary antibodies, membranes were scanned using the Odyssey CLx imaging system (LI-COR) or developed using CL-XPosure film (Thermo Fisher Scientific).

### Antibodies.

The various primary antibodies used for Western blotting in this study were: rabbit anti-GFP (1:3,000; TP401, Torrey Pines Biolabs), mouse anti-actin (1:5,000; 66009-1-lg, Proteintech), rabbit anti-cystinosin (1:1,000; in-house antibody), mouse anti-LAMP2 (1:1,000; H4B4, Developmental Studies Hybridoma Bank [DHSB]), rabbit anti-CTSD (1:1,000; 2284, Cell Signaling Technology), Tom20 (1:1,000; SC-17764, Santa Cruz Biotechnology), HRD1 (1:2,000; 134723-1-AP, Proteintech), Gapdh(1:10,000; 60004-1-Ig, Proteintech) VDAC 1/2 (1:1,000; 10866-1-AP, Proteintech), rabbit anti-HA (1:1,000, 715500, Life technologies), rabbit anti-p97 (1:1,000, a gift from Dr. Yanzhuang Wang**,** University of Michigan). The following secondary antibodies were used in this study: goat anti-mouse IRDye680LT(1:10,000, 92668020, Licor), goat anti-mouse IRDye800CW (1:10,000, 92632210, Licor), goat anti-rabbit IRDye680LT (1:10,000, 92632211, Licor), goat anti-rabbit IRDye800CW (1:10,000, 92632210, Licor), anti-Rabbit HRP (1:2000; JI 111-035-003, Jackson Immunoresearch).

All blots were blocked for 30 minutes in 5% milk (dissolved in 1 × TBST, TBST: 20 mM Tris pH 7.5, 150 mM NaCl, 0.1% Tween-20) at room temperature and incubated in primary antibody overnight at 4^^o^^C. They were washed 6 times, 5 minutes per wash. Blots were then incubated with secondary antibody for 1 hour at room temperature and washed 6 times, 5 minutes per wash. Blots were then developed.

### Immunofluorescence.

Cells were grown on circular glass coverslips, 12mm in diameter and 0.16–0.19mm thick. The different steps of immunostaining were performed in the dark by covering the coverslips with aluminum foil. Prior to fixation, cells were washed with ice-cold 1 × PBS. Subsequently, cells were fixed in cold 100% methanol for 8 minutes at –20°C. The fixed samples were blocked in 3% BSA (dissolved in 1 × PBS) for 1 hour at room temperature, followed by incubating with primary (2 hours, room temperature) and secondary antibodies (1 hour, room temperature). The nucleus was stained using Hoechst. Coverslips were mounted in Fluoromount-G (SouthernBiotech) and cured overnight before imaging. The following primary antibody was used for immunostaining in this study: mouse anti-LAMP2 (1:100, H4B4, DHSB). The following secondary antibody was used in this study: Cy-5 goat anti-mouse (1:100, JI 115-175-166 [Jackson Immunoresearch]).

### Microscopy and image processing.

Samples were imaged with a DeltaVision Elite system (GE Healthcare Life Sciences). The DeltaVision microscope was equipped with a scientific CMOS camera and an Olympus UPLXAP0100X objective. The filter sets FITC (excitation, 475/28; emission, 525/48), TRITC (excitation 542/27; emission 594/45), DAPI (excitation 390/18; emission 435/48), and cy-5 (excitation 632/22; emission 679/34), were used for GFP, mCherry, DAPI, and far-red, respectively. Image acquisition and deconvolution were performed with the softWoRx program. Images were further cropped or adjusted using ImageJ (National Institutes of Health).

### Ubiquitin blot.

Ubiquitin was N-terminally tagged with Hemagglutinin (HA) and the plasmid containing HA-Ubiquitin was transfected into a 10 cm dish of HEK293 cells expressing either cystinosin(WT)-GFP or cystinosin(7Δ)-GFP. After48 hours posttransfection, cells were harvested in ice-cold PBS and pelleted at 1,000*g* for 1 minute. The pellet was lysed in 350 μL of lysis buffer (20 mM Tris pH 8.0, 150 mM NaCl, 1% Triton, 1× PIC) containing 100 mM of N-Ethylmaleimide in 4°C for 20 minutes. Next, lysates were centrifuged at 18,000*g* for 15 minutes at 4°C and the supernatant was collected. Protein concentration of supernatants was measured using the Bradford assay, and all supernatants were normalized to equal protein concentration. 300 μL of normalized lysates were added to 25 μL of GFP-TrapA (preequilibrated with lysis buffer; Chromotek) and let to incubate overnight at 4°C. The resin was washed 3 times with lysis buffer and heated at 65^^o^^C with 2 × urea sample buffer (150 mM Tris pH 6.8, 6 M urea, 6% SDS, 40% glycerol, 100 mM DTT, 0.1% Bromophenol blue) at 65°C for 10 minutes to elute bound proteins. Eluates were then analyzed by Western blotting. If endogenous ubiquitin was analyzed, the same protocol was followed with the modification of boiling the nitrocellulose membrane for 30 minutes after the transfer of proteins.

### Lyso-IP.

The Lyso-IP protocol from Abu-Remaileh et al. (26) was modified to reduce ER and mitochondrial contamination from the immunoprecipitated lysosomes. A fully confluent 15 cm dish of HEK293 cells was used for one IP reaction. These cells were stably expressing TMEM192-3×HA and cystinosin(7Δ)-GFP. Cells were washed twice with ice-cold 1 × TBS (20 mM Tris pH 7.5, 150 mM NaCl) and harvested using a cell scraper in 1 mL TBS and pelleted at 1000*g* for 1 minute at 4°C. Each cell pellet was resuspended in 1 mL of 1 × TBS + protease inhibitor cocktail (Biotool) and homogenized with a Dounce homogenizer for 20 strokes. CaCl__2__ solution was added to the homogenates to a final concentration of 8 mM and homogenates were centrifuged at 1,150*g* for 3 minutes at 4°C. This step precipitates out a large portion of ER and mitochondria, leading to higher purity of lysosome yield in the IP fraction.

A total of 50 μL of the supernatant was taken from the previous centrifugation step and mixed with an equal volume of 2 × urea sample buffer to serve as the 5% input. The remaining 950 μL of the supernatant was mixed with 20 μL of magnetic anti-HA beads (Millipore-Sigma) (Preequilibrated with 1 × TBS + PIC + 8 mM CaCl__2__) for 45 minutes at 4°C. The beads were washed 3 times with IP buffer (1 × TBS + PIC + 8 mM CaCl__2__), and bound proteins were extracted by boiling the beads with 100μL of 1 × urea sample buffer. IP products were further analyzed via Western blot.

### Flow cytometry analysis.

Cells were washed with 1 × PBS and trypsinized until all cells were dissociated from the dishes. Dissociated cells were neutralized with DMEM containing 10% serum and pelleted at 300*g* for 3 minutes. Cells were resuspended in ice-cold 1 × PBS and analyzed using either an LSR Fortessa (BD Biosciences) or a Ze5 (Bio-Rad) flow cytometer. Flow cytometry analysis was performed by technicians from the Flow Cytometry Core at the University of Michigan. The data were analyzed using FlowJo software.

### In vitro deglycosylation.

A confluent 3.5 cm dish of HEK293 cells was taken as the starting material. Cells were rinsed once with 1 × ice-cold PBS and then harvested in 1 mL of PBS, split into 3 equal parts and centrifuged at 1,000*g* for 1 min at 4°C. Of the 3 pellets, 2 were resuspended in 40 μL of lysis buffer (20 mM Tris pH 8.0, 150 mM NaCl, 1% Triton, 1 × PIC), while the third pellet was resuspended in 1 × glyco-buffer 2 (50 mM sodium phosphate pH 7.5, 1% NP-40, 1 × PIC). All pellets were allowed to lyse for 20 minutes at 4°C. Lysates were subsequently centrifuged at 18,000*g* for 15 minutes at 4°C, and the protein concentration of the resulting supernatants was measured using Bradford assay. All supernatants were normalized to equal protein concentrations. One lysate in the lysis buffer was used as an untreated control. To the second lysate in lysis buffer, 3.9 μL of 10 × denaturation buffer (5% SDS, 400 mM DTT) was added to 35 μL of lysate and boiled at 98°C for 10 minutes. The lysate was then allowed to cool to room temperature for 5 minutes, and a further 4.7 μL of 10 × glyco-buffer 3 (500 mM sodium acetate pH 6) and 2μl of EndoH enzyme were added and incubated at 37°C overnight. To the lysate in glyco-buffer 2, 10 μL of glyco-buffer 2 was added to 35 μL of the lysate, and 2 μL of PNGase F was further added to it and incubated at 37°C overnight. After incubation, all lysates were mixed with an equal volume of 2 × urea sample buffer, heated at 65°C for 5 minutes, and analyzed by Western blot.

### Membrane isolation.

For isolating membrane fractions, 3 confluent 15 cm dishes were harvested. Cells were washed twice with ice-cold PBS and harvested by centrifugation at 2,500*g* for 1 minute at 4°C. Cells were resuspended in 1,000 μL homogenizing buffer (PBS + 1 × PIC + 1 mM EDTA + 1 mM EGTA) and homogenized using a Dounce homogenizer with 200 strokes. Homogenates were centrifuged at 900*g* for 5 minutes at 4°C to remove nuclear and mitochondrial fractions. The supernatant was taken into fresh tubes and centrifuged at 20,000*g* for 20 minutes at 4°C. Soluble fractions remain in the supernatant, while membrane fractions settle in the pellet. The supernatant was discarded, and pellets were lysed with lysis buffer (20 mM Tris pH 8.0, 150 mM NaCl, 1% Triton) containing 1 × PIC and processed for Western blotting.

### Cytosol-membrane separation.

A confluent 10 cm dish of HEK293 was harvested in ice-cold PBS and centrifuged at 1,000*g* for 1 minute at 4^^o^^C. Cells were resuspended in 1 mL of modified HB1 lysis buffer (20 mM HEPES-KOH pH 7.2, 100 mM Sucrose, 1 mM EDTA + 1 × protease inhibitor cocktail), and cells were passed through a 22 G needle 50 times. 10% homogenate was taken as input and heated at 65°C with 2 × urea sample buffer. The remaining volume of homogenates was ultra-centrifuged at 100,000*g* for 30 minutes at 4°C. The supernatants were collected into a fresh tube, the pellet was resuspended in 1 mL modified HB1 lysis buffer, and both supernatants and pellets were precipitated with 10% TCA at 4°C overnight. Following TCA precipitation, protein pellets were obtained by centrifugation at 18,000*g* for 5 minutes and further heated at 65°C with 2 × urea sample buffer. All samples were analyzed using Western blotting.

### ER isolation using Optiprep density gradient.

Optiprep density gradient was set up in increasing steps from 10% to 32% (please see Supplemental Table 4) by diluting 60% Optiprep (Sigma-Aldrich) in sucrose buffer (0.25 M sucrose, 1 mM EDTA, 10 mM Tris-HCl, pH 7.4). A confluent 15 cm dish of HEK293 cells was harvested in ice-cold PBS and centrifuged at 1,000*g* for 1 minute at 4°C. Cell pellets were then resuspended in 600 μl extraction buffer (0.25 M sucrose, 10 mM triethanolamine, 10 mM acetic acid, pH 7.8) and lysed with a Dounce homogenizer. The cell lysates were layered on top of the density gradient and ultra-centrifuged at 150,000*g* at 4°C for 4 hours in an SW41Ti rotor. 1 mL fractions were collected into an individual tube and diluted with 9 mL water. To these diluted fractions, 1.1 mL of 100% TCA was added, and proteins were allowed to precipitate overnight at 4°C. Subsequently, the fractions were centrifuged at 18,000*g* for 5 minutes, protein precipitates were mixed with 2 × urea sample buffer and heated at 65°C for 5 minutes, and further analyzed via Western blotting.

### Live-cell imaging.

All live-cell movies were captured using Nikon CSU-X1 spinning disk confocal microscope. Movies were captured at 100 milliseconds, 5% laser power. Cells were constantly maintained at 37°C and 5% CO__2__ using the Tokai Hit system. Images were analyzed and deconvolved using NIS Elements software.

### Mass spectrometry to analyze lysosomal cystine levels.

Human fibroblasts listed in Supplemental Table 2 were cultured in DMEM with 10% FBS plus penicillin and streptomycin. For steady cystine levels, fibroblasts were cultured in 10 cm dishes until they reached 80% confluency (Figure 7B). For the chemical chaperone experiments in Figure 7D, C3 and C4 were added at 60% confluency and treated for 72 hours. Cells were harvested by trypsinization, and pellets were washed twice in DPBS (Gibco). Approximately 1 × 10^^6^^ cells were homogenized in 150 μL 5 mM NEM solution for 20 strokes. 50μl of 15% SSA (sulfosalicylic acid) solution was added to the homogenates, mixed, and centrifuged at 13,500 × g for 10 minutes at 4°C. Supernatants were collected into fresh tubes, and the volume was made up to 500 μL with 3% SSA and snap frozen with liquid nitrogen. Frozen samples were shipped to UCSD biochemical genetics laboratory to analyze cystine levels through mass spectrometry. Pellets from centrifugation were dissolved in 0.1 N NaOH, and the protein amount was assayed by BCA assay to estimate the total protein amount.

### In vitro deubiquitinase assay.

Cells expressing cystinosin (Δ7)-GFP were treated with either DMSO, BafA1, or MG132 for 6 hours. After treatment, cells were lysed with lysis buffer (20 mM Tris pH 8.0, 150 mM NaCl, 1% Triton, 1 × PIC), and treated with recombinant His8-USP7 (E-519-025, Bio-techne) at a final concentration of 20 nM for 6 hours at 37°C. The enzymatic reaction was terminated by adding 2 × urea Buffer (150 mM Tris pH 6.8, 6 M urea, 6% SDS, 40% glycerol, 100 mM DTT, 0.1% bromophenol blue) and heating the samples at 65°C for 5 minutes. Samples were resolved by SDS PAGE gel and probed with either Anti-GFP or Anti-Ubiquitin antibodies.

### Statistics.

The band intensity for the Western blot was quantified using Image Studio software (LI-COR). Statistical analysis was performed with ordinary 1-way ANOVA. Error bars represent the SD. A *P* value less than 0.05 was considered significant.

### Study approval.

All procedures were approved by and done in accordance with the University of Michigan, approval number IBCA00000369.

### Data availability.

All uncropped original blots are submitted to the editor and available upon request. All quantifications are provided in the Supporting Data Values file.

## Author contributions

VV and ML conceptualized the study. VV, ML, and JT developed the methodology. VV, WZ, and XY performed the investigation. SHH provided critical reagents. VV, XY, and ML wrote and edited the manuscript. ML and SH acquired funding for the study. ML provided resources and supervision.

## Supplementary Material

Supplemental data

Supplemental video 1

Supplemental video 2

Supplemental video 3

Supplemental video 4

Supplemental video 5

Supporting data values

## Version 1 08/10/2023

In-Press Preview

## Version 2 10/02/2023

Electronic publication
