# Supplementary material for: ER-associated degradation in cystinosis pathogenesis and the prospects of precision medicine
Source: J Clin Invest. 2023 Oct 2;133(19):e169551. doi: 10.1172/JCI169551 (PMC10541201; doi:10.1172/JCI169551)
Supplement: Supplemental data [file jci-133-169551-s166.pdf]

Inventory of Supplemental Information

ER-associated degradation in cystinosis pathogenesis and the prospects of precision medicine

Varsha Venkatarangan, Weichao Zhang, Xi Yang, Jess Thoene, Si Houn Hahn, and Ming Li

I. Supplemental Figure Data

a. Supplemental Figure 1

b. Supplemental Figure 2

c. Supplemental Figure 3

d. Supplemental Figure 4

II. Supplemental Table

a. Supplemental Table 1

b. Supplemental Table 2

c. Supplemental Table 3

d. Supplemental Table 4

III. Supplemental Movies

a. Supplemental Movie 1

b. Supplemental Movie 2

c. Supplemental Movie 3

d. Supplemental Movie 4

e. Supplemental Movie 5

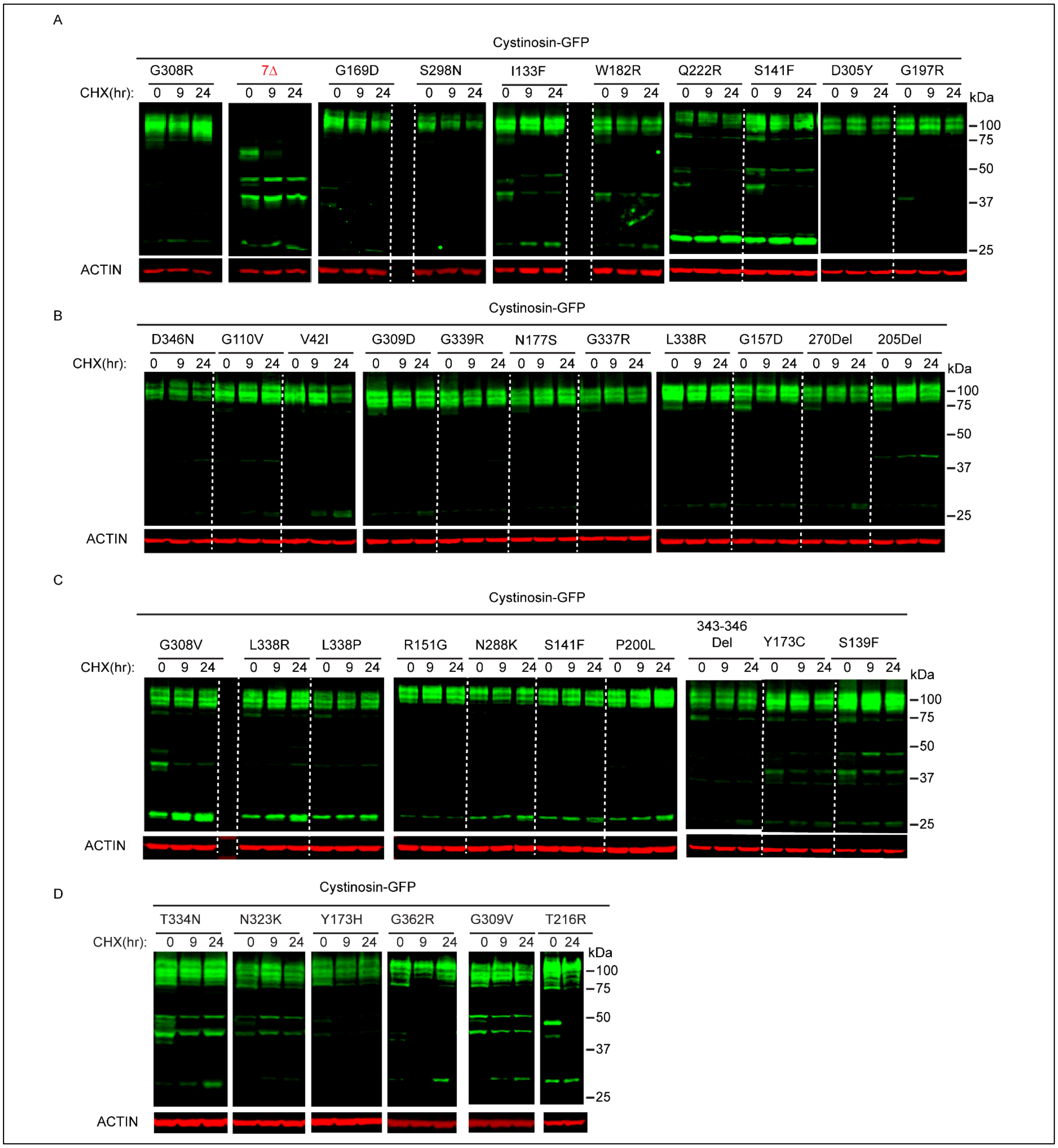

Figure S1: Screening of the different cystinosin disease mutants

(A-D) HEK293 cells transiently expressing different cystinosin patient mutants were treated with cycloheximide for indicated times and analyzed with immunoblotting.

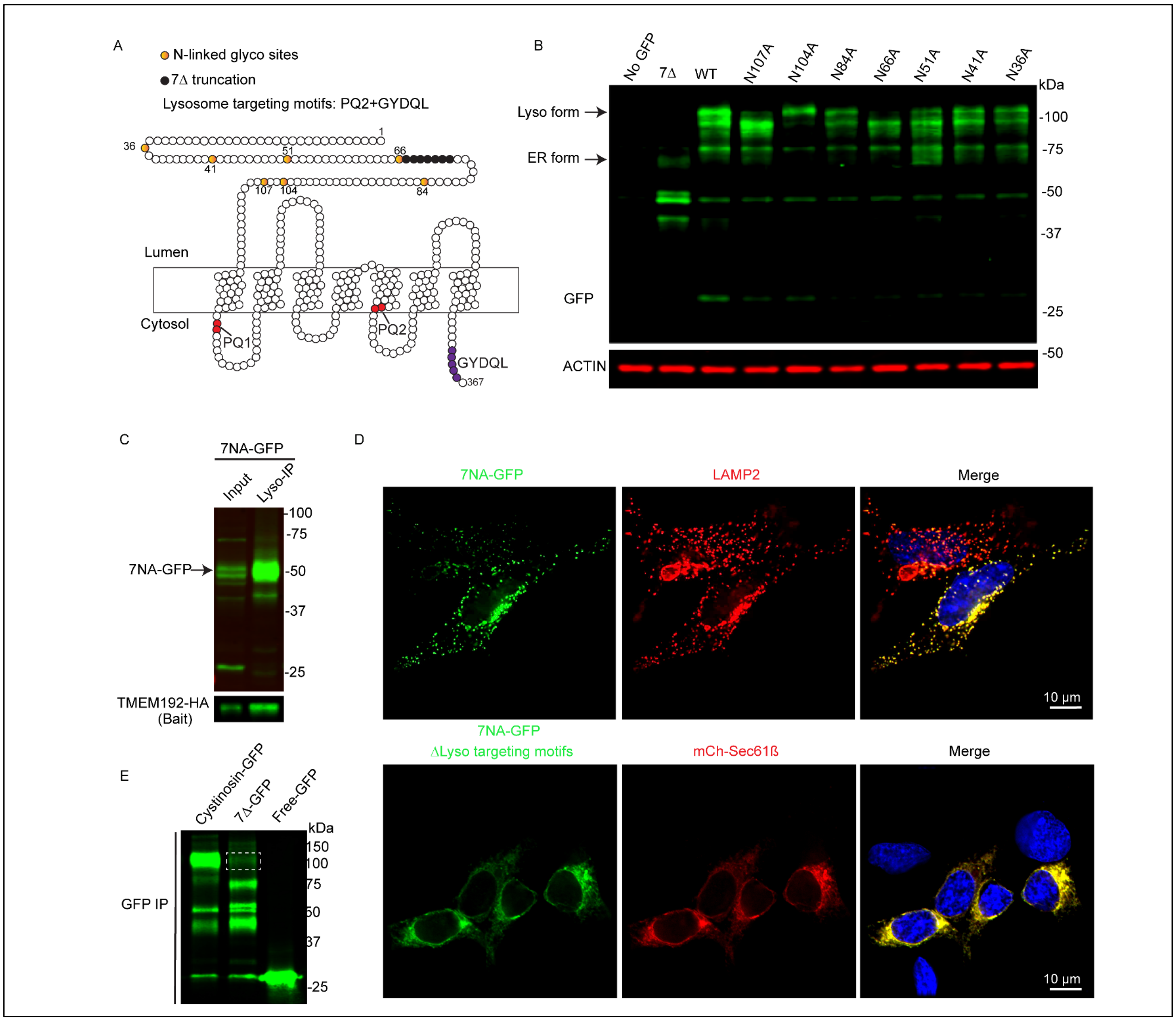

Figure S2: N-linked glycosylation is not required for the ER exit of cystinosin, but the lysosome targeting motifs are.

(A) Topology map showing all 7 putative glycosylation sites, 2 PQ motifs, and the GYDQL motif. **(B)** Analysis of individual N-linked glycosylation mutants. All mutants can exit the ER and mature into the lysosome form, unlike cystinosin(7Δ)-GFP. **(C)** Lyso-IP to verify that cystinosin(7NA)-GFP is enriched in the lysosome fraction. **(D)** The lysosome localization of cystinosin(7NA)-GFP depends on the lysosomal targeting motifs, including PQ2 and GYDQL. **(E)** Immunoprecipitation of WT cystinosin-GFP and cystinosin(7Δ)-GFP revealed that cystinosin(7Δ)-GFP has a small portion of lysosome form, highlighted with a white box.

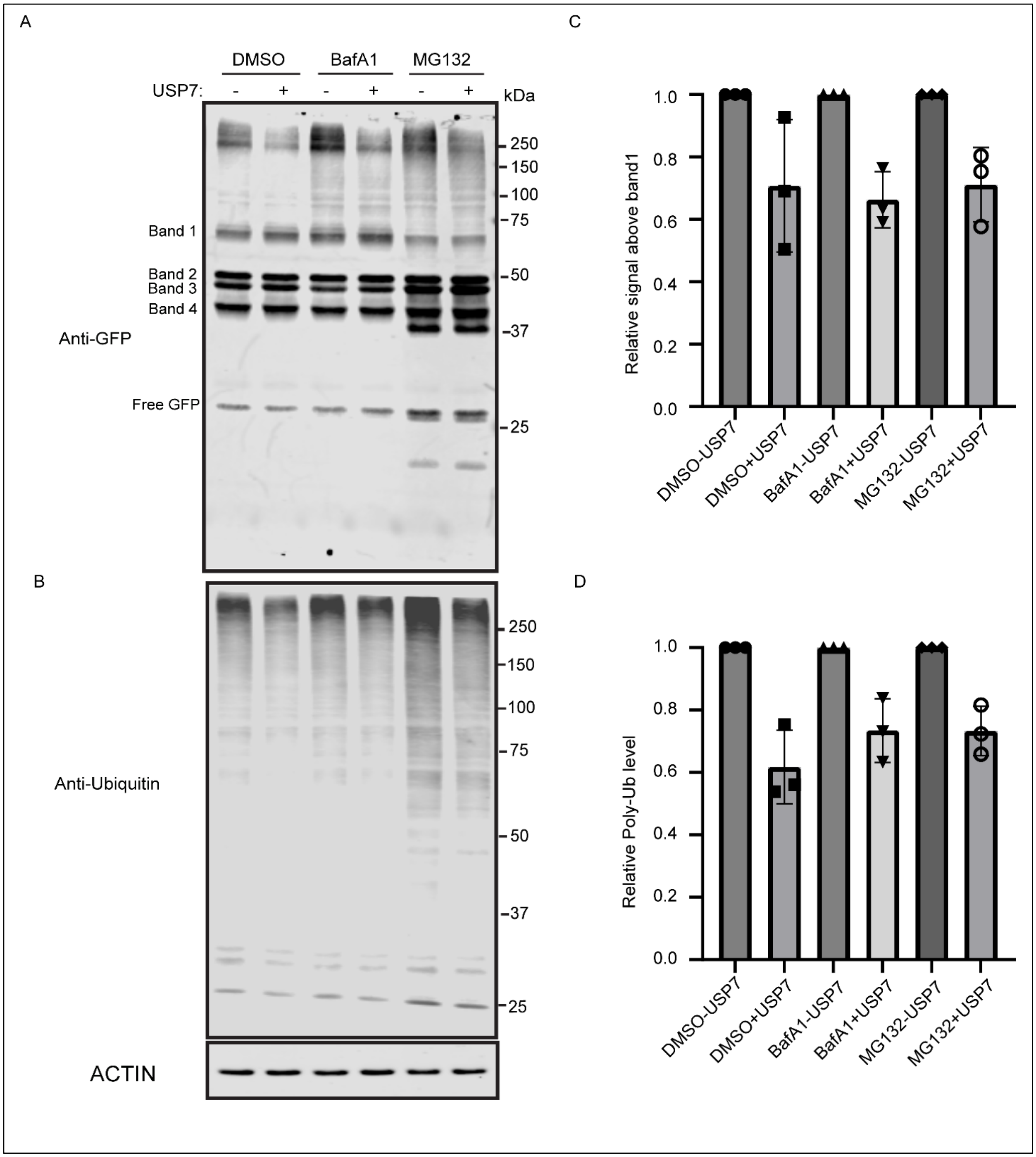

Figure S3: Partial reduction of higher-molecular-weight species by USP7 treatment. (A-B) Whole cell lysates from DMSO, BafA1, and MG132-treated samples were subjected to subsequent treatment with USP7 for 6 hours at 30°C and then probed with anti-GFP (A) or anti-Ubiquitin antibodies (B). (**C-D)** Quantification of the results shown in panels A and B, respectively. Data represent mean ± STDEV from 3 independent replicates.

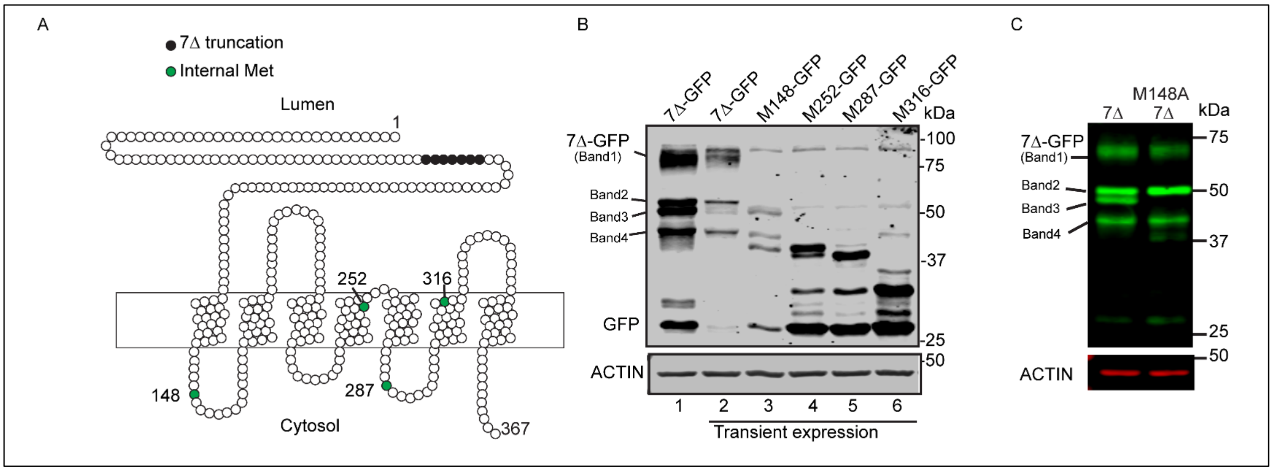

Figure S4: Band 3 is an internal translation-initiation product from methionine^{148}

(A) Topology map showing the different methionines (marked in green). **(B)** Size comparison between cystinosin(7Δ)-GFP and truncation products starting from internal methionines. **(C)** M148A mutation abolished band 3 in cystinosin(7Δ)-GFP.

Supplemental Table1: Cell lines used in this study

Cell lines	***Description***	***reference/source***
Human HEK293	CRL-1573	ATCC
Human HEK293T	CRL-3216	ATCC
Human HeLa	CCL-2	ATCC
Human HEK293, Cystinosin-GFP	pHAGE2-EF1α-CTNS-EGFP-IRES-Puro	This study
Human HEK293, Cystinosin(∆7)-GFP	pHAGE2-EF1α-CTNS(7∆)-EGFP-IRES-Puro	This study
Human HEK293, TMEM192-3XHA, Cystinosin(∆7)-GFP	pLJC5-TMEM192-3XHA-Puro (Addgene 102930), pHAGE2-EF1α-CTNS(7∆)-EGFP-IRES-Hygro	This study (Abu-Remaileh et al. 2017)
Human HEK293, Cystinosin(∆7)	pHAGE2-EF1α-CTNS(7∆)-IRES-Blasticidin	This study
Human HEK293, Cystinosin-GFP	pCW57.1-CTNS-GFP-Puro	This study
Human HeLa, Cystinosin-GFP	pHAGE2-EF1α-CTNS-EGFP-IRES-Puro	This study
Human HeLa, Cystinosin(∆7)-GFP	pHAGE2-EF1α-CTNS(7∆)-EGFP-IRES-Puro	This study
Human HeLa, Cystinosin(7NA ∆Lyso targeting motif)-GFP	pHAGE2-EF1α-CTNS(7NA ∆Lyso targeting motif)EGFP-IRES-Puro	This study
Hrd1 CRISPR-Cas9 KO,<br><br>Cystinosin(∆7)-GFP	CRISPR-Cas9 KO of Hrd1, single colony, pHAGE2-EF1α-CTNS(7∆)-EGFP-IRES-Puro	This study
Human HEK293, Cystinosin(∆7+M148A)-GFP	pHAGE2-EF1α-CTNS(7∆+M148A)-EGFP-IRES-Puro	This study

Human HEK293, TMEM192-3XHA, Cystinosin(7NA)-GFP	pLJC5-TMEM192-3XHA-Puro (Addgene 102930), pHAGE2-EF1α-CTNS(7NA)-EGFP-IRES-Hygro	This study
Human HEK293, TMEM192-3XHA, Cystinosin(Δ7+7NA)-GFP	pLJC5-TMEM192-3XHA-Puro (Addgene 102930), pHAGE2-EF1α-CTNS(7Δ+7NA)-EGFP-IRES-Hygro	This study
Human HEK293, Cystinosin(N36A)-GFP	pHAGE2-EF1α-CTNS(N36A)-EGFP-IRES-Puro	This study
Human HEK293, Cystinosin(N41A)-GFP	pHAGE2-EF1α-CTNS(N41A)-EGFP-IRES-Puro	This study
Human HEK293,<br><br>Cystinosin(N51A)-GFP	pHAGE2-EF1α-CTNS(N51A)-EGFP-IRES-Puro	This study
Human HEK293, Cystinosin(N66A)-GFP	pHAGE2-EF1α-CTNS(N66A)-EGFP-IRES-Puro	This study
Human HEK293, Cystinosin(N84A)-GFP	pHAGE2-EF1α-<br><br>CTNS(N84A)-EGFP-IRES-Puro	This study
Human HEK293, Cystinosin(N104A)-GFP	pHAGE2-EF1α-CTNS(N104A)-EGFP-IRES-Puro	This study
Human HEK293, Cystinosin(N107A)-GFP	pHAGE2-EF1α-CTNS(N107A)-EGFP-IRES-Puro	This study

Supplemental Table 2: Patient fibroblasts used in this study

Cell lines	***Description***	***reference/source***
Healthy fibroblasts	GM05658<br><br>Fibroblasts from a healthy individual.	Coriell
Cystinotic fibroblasts	GM00706	Coriell

	Fibroblasts from an individual harbouring a homozygous 57kBp genomic DNA deletion.	
Cystinotic fibroblasts	Fibroblasts from an individual harbouring a homozygous 21 base pair deletion (7Δ).	Dr. William Gahl, NIH(Shotelersuk, Larson et al. 1998)

Supplemental Table 3: Mammalian plasmids used in this study

Vector	***Insert***	***description***	***reference/source***
pHAGE2-IRES-Puro	CTNS-EGFP	EF1α promoter, Puro	This study
pHAGE2-IRES-Puro	CTNS(7Δ)-EGFP	EF1α promoter, Puro	This study
pHAGE2-IRES-Blasticidin	CTNS(7Δ)-EGP	EF1α promoter, Blasticidin	This study
pmCherry C1	Sec61β (Mouse)	CMV promoter	This study
pLJC5	TMEM192-3XHA	UbC promoter	Addgene 102930(Abu-Remaileh, Wyant et al. 2017)
pHAGE2-IRES-Hygro	CTNS(7Δ)-EGFP	EF1α promoter, Hygro	This study
pHAGE2-IRES-Blasticidin	CTNS(7NA ΔLyso targeting motif)-EGFP	EF1α promoter, Blasticidin	This study
pSpCas9(BB)-2A-Puro (PX459)	sgRNA for Hrd1	CRISPR-Cas9 knockout	Addgene, 48139(Ran, Hsu et al. 2013)
pmCherry N1	Sar1	CMV promoter	This study
pmCherry N1	Sar1	CMV promoter	This study

pCW57.1	CTNS-EGFP	TRE promoter	This study
pEGFPN1	CTNS(7Δ)	CMV promoter	This study
pEGFPN1	CTNS(M148 start)	CMV promoter	This study
pEGFPN1	CTNS(M252 start)	CMV promoter	This study
pEGFPN1	CTNS(M287 start)	CMV promoter	This study
pEGFPN1	CTNS(M316 start)	CMV promoter	This study
psPAX2		Lentiviral packaging plasmid	Addgene 12260
pMD2.G		VSV-G envelope	Addgene 12259
pHAGE2-IRES-Puro	CTNS(N36A)-EGFP	EF1α promoter, Puro	This study
pHAGE2-IRES-Puro	CTNS(N41A)-EGFP	EF1α promoter, Puro	This study
pHAGE2-IRES-Puro	CTNS(N51A)-EGFP	EF1α promoter, Puro	This study
pHAGE2-IRES-Puro	CTNS(N66A)-EGFP	EF1α promoter, Puro	This study
pHAGE2-IRES-Puro	CTNS(N84A)-EGFP	EF1α promoter, Puro	This study
pHAGE2-IRES-Puro	CTNS(N104A)-EGFP	EF1α promoter, Puro	This study
pHAGE2-IRES-Puro	CTNS(N107A)-EGFP	EF1α promoter, Puro	This study
pEGFPN1	CTNS(G308R)	CMV promoter	This study
pEGFPN1	CTNS(G169D)	CMV promoter	This study
pEGFPN1	CTNS(S298N)	CMV promoter	This study
pEGFPN1	CTNS(I133F)	CMV promoter	This study

pEGFPN1	CTNS(W182R)	CMV promoter	This study
pEGFPN1	CTNS(Q222R)	CMV promoter	This study
pEGFPN1	CTNS(S141F)	CMV promoter	This study
pEGFPN1	CTNS(D305Y)	CMV promoter	This study
pEGFPN1	CTNS(G197R)	CMV promoter	This study
pEGFPN1	CTNS(D346N)	CMV promoter	This study
pEGFPN1	CTNS(G110V)	CMV promoter	This study
pEGFPN1	CTNS(V42I)	CMV promoter	This study
pEGFPN1	CTNS(G309D)	CMV promoter	This study
pEGFPN1	CTNS(G339R)	CMV promoter	This study
pEGFPN1	CTNS(N177S)	CMV promoter	This study
pEGFPN1	CTNS(G337R)	CMV promoter	This study
pEGFPN1	CTNS(L338R)	CMV promoter	This study
pEGFPN1	CTNS(G157D)	CMV promoter	This study
pEGFPN1	CTNS(Δ270)	CMV promoter	This study
pEGFPN1	CTNS(Δ205)	CMV promoter	This study
pEGFPN1	CTNS(G308V)	CMV promoter	This study
pEGFPN1	CTNS(L338P)	CMV promoter	This study
pEGFPN1	CTNS(R151G)	CMV promoter	This study
pEGFPN1	CTNS(N288K)	CMV promoter	This study
pEGFPN1	CTNS(S141F)	CMV promoter	This study
pEGFPN1	CTNS(P200L)	CMV promoter	This study
pEGFPN1	CTNS(Δ343-346)	CMV promoter	This study
pEGFPN1	CTNS(Y173C)	CMV promoter	This study

pEGFPN1	CTNS(S139F)	CMV promoter	This study
pEGFPN1	CTNS(T334N)	CMV promoter	This study
pEGFPN1	CTNS(N323K)	CMV promoter	This study
pEGFPN1	CTNS(Y173H)	CMV promoter	This study
pEGFPN1	CTNS(G362R)	CMV promoter	This study
pEGFPN1	CTNS(G309V)	CMV promoter	This study
pEGFPN1	CTNS(T216R)	CMV promoter	This study

Supplemental Table 4: Optiprep density gradient preparation

Preparation of increasing density fractions (10-32%) for organelle density fractionation in a 13.2mL open-top thin wall ultra-clear tube, 14x89mm. 60% OptiPrep™ solution was further diluted to a 50% OptiPrep™ using a sucrose buffer that contained 0.25M sucrose, 6mM EDTA, 60mM Tris-HCl, pH7.4. This 50% OptiPrep™ was further diluted into the different concentration percentages as mentioned in the table using a buffer called the dilution medium (0.25M sucrose, 1mM EDTA, 10mM Tris-HCl, pH7.4, 1x complete protease inhibitor cocktail) Fractions were layered in an ultra-centrifuge tube with the densest layer, 34% OptiPrep™, layered on the bottom of the tube all the way to the lightest layer, 10% OptiPrep™ layered on the top. Finally, the cell lysate was loaded on top of the gradient.(Bryant, Liu et al. 2018)

**Fraction Density (% OptiPrep)**	**50% OptiPrep™ Medium (µL)**	**Dilution Medium (µL)**
0 (Cell Lysate; Top)	N/A (600 µL Cell Lysate)	N/A
10	120	480
12	144	456
14	168	432

16	192	408
18	216	384
20	240	360
22	264	336
24	288	312
26	312	288
28	336	264
30	360	240
32	384	216
34 (Bottom)	3264	1536

Movie S1: WT Cystinosin-GFP is localized to lysosomes

Z-stack imaging through HeLa cells stably expressing WT cystinosin-GFP. Step size: 0.4μm

Movie S2: Cystinosin(7∆)-GFP is localized to both ER and lysosomes

Z-stack imaging through HeLa cells stably expressing cystinosin(7∆)-GFP. Step size: 0.4μm.

Besides punctae (lysosomes), a nuclear envelope (ER) signal was also observed.

Movie S3: A time-lapse movie of Cystinosin(7∆)-GFP treated with CHX

HeLa cells stably expressing cystinosin(7∆)-GFP were treated with CHX for 6 hours. Z-stack images were collected every hour during the treatment. Both fluorescence and DIC images were collected. At 6 hours, the nuclear envelope signal disappeared, but the punctate signal remained.

Movie S4: A time-lapse movie of Cystinosin(7∆)-GFP treated with vehicle

HeLa cells stably expressing cystinosin(7∆)-GFP were treated with vehicle (0.1% ethanol) for 6 hours. Z-stack images were collected every hour during the treatment. Both fluorescence and DIC images were collected. At 6 hours, the nuclear envelope signal was still visible.

Movie S5: A time-lapse movie of Cystinosin(7∆)-GFP treated with CHX and kifunensine

HeLa cells stably expressing cystinosin(7∆)-GFP were treated with CHX and kifunensine for 6 hours. Z-stack images were collected every hour during the treatment. Both fluorescence and DIC images were collected. At 6 hours, the nuclear envelope signal was still visible.

References:

1) Shotelersuk V, Larson D, Anikster Y, McDowell G, Lemons R, Bernardini I, et al. CTNS mutations in an American-based population of cystinosis patients. *Am J Hum Genet.* 1998;63(5):1352-62.
2) Abu-Remaileh M, Wyant GA, Kim C, Laqtom NN, Abbasi M, Chan SH, et al. Lysosomal metabolomics reveals V-ATPase- and mTOR-dependent regulation of amino acid efflux from lysosomes. *Science.* 2017;358(6364):807-13.
3) Ran FA, Hsu PD, Wright J, Agarwala V, Scott DA, and Zhang F. Genome engineering using the CRISPR-Cas9 system. *Nat Protoc.* 2013;8(11):2281-308.
4) Bryant D, Liu Y, Datta S, Hariri H, Seda M, Anderson G, et al. SNX14 mutations affect endoplasmic reticulum-associated neutral lipid metabolism in autosomal recessive spinocerebellar ataxia 20. *Hum Mol Genet.* 2018;27(11):1927-40.
